# The Pathophysiology of Degenerative Cervical Myelopathy and the Physiology of Recovery Following Decompression

**DOI:** 10.3389/fnins.2020.00138

**Published:** 2020-04-30

**Authors:** Farhana Akter, Xinming Yu, Xingping Qin, Shun Yao, Parisa Nikrouz, Yasir Ahmed Syed, Mark Kotter

**Affiliations:** ^1^Department of Clinical Neuroscience, University of Cambridge, Cambridge, United Kingdom; ^2^Faculty of Arts and Sciences, Harvard University, Cambridge, MA, United States; ^3^Massachusetts General Hospital Cancer Center, Harvard Medical School, Boston, MA, United States; ^4^Department of Neurosurgery, Brigham and Women's Hospital, Harvard Medical School, Boston, MA, United States; ^5^Maidstone and Tunbridge Wells Trust, Maidstone, United Kingdom; ^6^Neuroscience and Mental Health Research Institute (NMHRI), Cathays, United Kingdom; ^7^School of Bioscience, Cardiff University, The Sir Martin Evans Building, Cardiff, United Kingdom

**Keywords:** neuronal loss, apoptosis, pathogenesis, spine, degeneration

## Abstract

**Background:** Degenerative cervical myelopathy (DCM), also known as cervical spondylotic myelopathy is the leading cause of spinal cord compression in adults. The mainstay of treatment is surgical decompression, which leads to partial recovery of symptoms, however, long term prognosis of the condition remains poor. Despite advances in treatment methods, the underlying pathobiology is not well-known. A better understanding of the disease is therefore required for the development of treatments to improve outcomes following surgery.

**Objective:** To systematically evaluate the pathophysiology of DCM and the mechanism underlying recovery following decompression.

**Methods:** A total of 13,808 published articles were identified in our systematic search of electronic databases (PUBMED, WEB OF SCIENCE). A total of 51 studies investigating the secondary injury mechanisms of DCM or physiology of recovery in animal models of disease underwent comprehensive review.

**Results:** Forty-seven studies addressed the pathophysiology of DCM. Majority of the studies demonstrated evidence of neuronal loss following spinal cord compression. A number of studies provided further details of structural changes in neurons such as myelin damage and axon degeneration. The mechanisms of injury to cells included direct apoptosis and increased inflammation. Only four papers investigated the pathobiological changes that occur in spinal cords following decompression. One study demonstrated evidence of axonal plasticity following decompressive surgery. Another study demonstrated ischaemic-reperfusion injury following decompression, however this phenomenon was worse when decompression was delayed.

**Conclusions:** In preclinical studies, the pathophysiology of DCM has been poorly studied and a number of questions remain unanswered. The physiological changes seen in the decompressed spinal cord has not been widely investigated and it is paramount that researchers investigate the decompressed spinal cord further to enable the development of therapeutic tools, to enhance recovery following surgery.

## Introduction

Degenerative cervical myelopathy (DCM), previously known as cervical spondylotic myelopathy (CSM) is a degenerative condition of the spine leading to mechanical compression of the spinal cord. Despite the prevalence of this condition, the pathogenesis underlying mechanical stress induced injury is poorly understood. Interestingly, to complicate matters further, the degree of compression of the spinal cord is not related to the severity of cord compression (Boden et al., 1990; Lebl et al., 2011). DCM can occur due to both static and dynamic stressors. The former is related to stenosis of the developmental canal, intervertebral disc bulging, and hypertrophy of the ligamentum flavum. Dynamic stressors include invagination of the ligamentum flavum (Nishida et al., 2012). Histopathological analysis of DCM suggests that the disease is mainly confined to the white matter tracts with evidence of wallerian degeneration of motor axons in the lateral corticospinal tract (McMinn, 1998), leading to clinical symptoms such as spastic gait. Patients who suffer with symptoms of poor sensation, proprioception defects or sphincter disturbance typically have degeneration of the central gray matter and posterior column (Lunsford et al., 1980). The underlying mechanism of injury includes cell death, increased inflammation and myelin damage. However, the exact pathophysiological mechanisms of DCM are not fully understood, thereby limiting our ability to develop therapeutics that can be used to improve prognosis. Most patients with DCM are treated with surgery, which can halt disease progression. However, many patients relapse and the long term outcomes are poor. In this review, we discuss the findings of our systematic analysis of the available literature to enable us to understand the pathobiology of this condition during compression and also the cellular and structural changes that take place following decompression.

## Objectives

To systematically review the literature on the pathophysiology of DCM and physiology of recovery following surgical decompression.

Research questions:

What is the pathophysiology of DCM?
What are the physiological and pathological changes following decompression?

## Methods

An electronic search of the literature was performed by two reviewers (F.A and X.Y) to identify all studies, on the pathophysiology of DCM.

The MEDLINE (January 1955 to February 2019) AND Web Of Science (January 1900 to December 2019) databases were searched by using a sensitive search strategy (Leenaars et al., 2012) that combined medical subject headings and terms with free text words in Ovid; these terms were the following:

[(“cervical spondylotic myelopathy” OR “cervical myelopathy” OR “spinal cord compression” OR “spinal cord decompression” OR “degenerative cervical myelopathy”)] AND (“pathogenesis” OR “pathology” OR “pathobiology” OR “biology” OR “inflammation” OR “apoptosis” OR “ischemia” OR “demyelination” OR “axon loss”).

Two reviewers (F.A and X.Y) independently assessed the titles and key words of all eligible citations to determine if the studies met our inclusion criteria. If the content of a study was not obvious from the title and key words, the abstract was retrieved and evaluated by both reviewers for eligibility. In the second step, abstracts of articles that were eligible for inclusion in the study were reviewed independently. We also screened the reference lists of the included papers for further studies. Finally, the original studies of the selected articles were evaluated independently (F.A and X.Y). At any stage, disagreements were discussed and resolved in a consensus meeting with the senior author before the next step could be performed.

### Inclusion Criteria (See Table 1)

This systematic review includes papers, which investigated the pathophysiology of DCM in pre-clinical models. Specifically, we included studies, which reported on the cellular and the mechanisms of injury seen in DCM. We included studies involving both *in vitro* models and *in vivo* models.

**Table 1:** Inclusion and exclusion criteria.

**Inclusion criteria**	**Exclusion criteria**
English language	Human studies
Experimental pre-clinical animal *in vitro* models of chronic spinal cord compression	Acute spinal cord compression models
Experimental pre-clinical animal *in vivo* models of chronic spinal cord compression	Lesions induced below the 7th cervical vertebra
Studies investigating the pathogenesis of degenerative cervical myelopathy	Reviews
Studies investigating the physiology of recovery following decompression of degenerative cervical myelopathy	Conference proceedings

Excluded were studies, which looked at acute spinal cord injury or compression, reviews, and spinal cord injuries induced at levels other than the cervical region. We also excluded single case reports and conference proceedings. Articles written in languages other than English were excluded, however we did not restrict our search to any particular geographical region.

### Data Extraction

Data extraction and quality assessment was performed independently by the two investigators using “The Animals in Research: Reporting *in vivo* Experiments (ARRIVE)” Guidelines. The design and methodology of individual studies were extracted including the type of study, sample size, experimental groups, animal housing, and length of follow-up. Species of animal as well as age, strain, weight, sex, and genetic modification factors were extracted. These results were entered into an Excel spreadsheet and studies were assessed for heterogeneity and quality.

### Assessment of Risk of Bias and Study Quality

Risk of bias was assessed by two authors. Discrepancies between authors were resolved by discussion with the senior author (MK) until consensus was achieved. The Systematic Review Center for Laboratory animal Experimentation (SYRCLE) risk of bias tool, which includes domains for selection bias (sequence generation, baseline characteristics, allocation concealment), performance bias (random housing and blinding), detection bias (random outcome assessment and blinding), attrition bias, reporting bias, and other biases was used. Studies were subsequently graded as “low,” “moderate,” or “high” according to quality. This grading method was designed by the authors to provide an overall quality rating following an assessment of the overall methodology of the study. Studies were graded as of “high” quality, when two or more methods were used to cross validate any findings and thus increasing the confidence that the stated conclusions of the paper were more likely to be a true effect. If a study was conducted using a robust methodology but not appropriately quantified or cross validated, it was downgraded to “moderate.” Studies were further downgraded to “low” if there were significant and unexplained variability in results or conclusions were made using simple observational methods without any cross-validation. There is a considerable amount of subjectivity using this method and thus any differing conclusions made by the primary authors were discussed with the senior author to help achieve a consensus regarding the quality of the paper.

## Results

**Figure 1:**
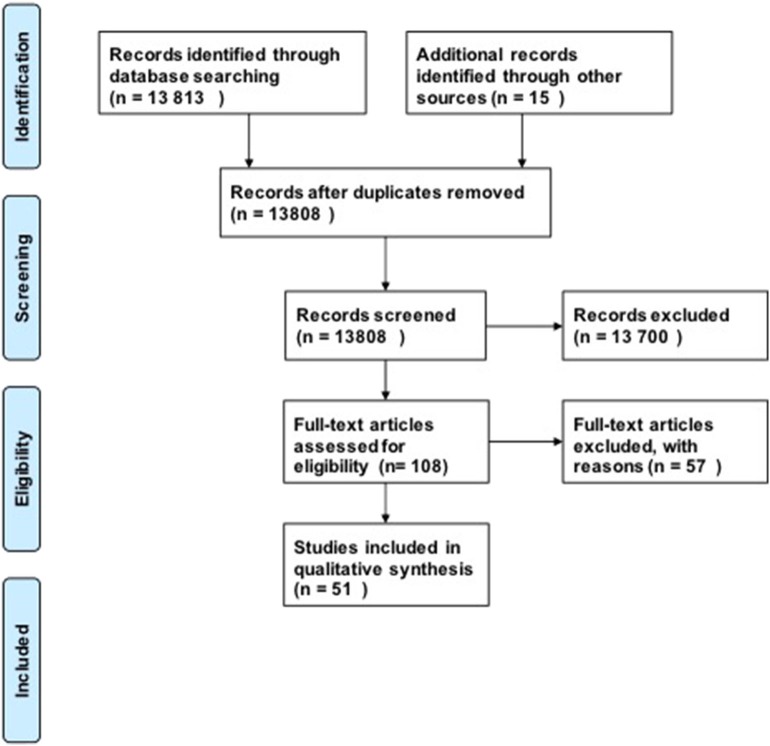
PRISMA Flow diagram.

For our research questions, we included 51 studies in our analysis using our search strategy (Figure 1). Our initial search produced 13,808 possible publications for review for these three key questions. After title and abstract review, we excluded 13,700 publications, the majority of which did not discuss DCM or were review studies. We found 15 additional publications from the references in-text, giving a total of 108 publications for full-text review. After full-text review, 57 were excluded for the following reasons: human studies (*n* = 8), compression in thoracic level (*n* = 12), compression in lumbar region (*n* = 1), not in English language (*n* = 4), paper did not investigate the pathophysiology of DCM or physiology of recovery (*n* = 32). Fifty-one studies were subsequently included in the quantitative analysis. The full evidence summary for these included studies can be found in Tables S1–S3. We used our grading method grade the quality of evidence found (Table 2).

**Table 2:** Summary of pathophysiological changes following chronic spinal cord compression.

**Findings**	**Strength of evidence**	**References**
**Cell response**		
Neuron loss at the epicenter of compression	Moderate- poor Most studies did not cross validate findings. However, a number of studies used two markers for a single approach e.g., IH.	Yamaguchi, 1980; Shinomiya et al., 1992; Baba et al., 1996; Yato et al., 1997; Uchida et al., 2002, 2008; Yamaura et al., 2002; Mallik and Weir, 2005; Zhao et al., 2005; Kadota et al., 2012; Lee et al., 2012; Aung et al., 2013; Hirai et al., 2013; Karadimas et al., 2013a, 2015; Bathina and Das, 2015; Cheng et al., 2015; Dhillon et al., 2016; Yoshizumi et al., 2016
Oligodendrocyte loss	Poor Most studies used a single approach with one marker ± no quantification	Liu et al., 2000; Zhao et al., 2005; Takenouchi et al., 2008; Hu et al., 2011; Moon et al., 2014
Astrogliosis at compressed site or distal to compressed site	Poor Single approach with no cross validation One marker of astrogliosis	Hukuda and Wilson, 1972; Ozawa et al., 2004; Yu et al., 2009, 2011; Moon et al., 2014; Dhillon et al., 2016
**Structural changes**		
Vascular insufficiency in spinal arteries	Moderate Limitations: Cross-validation with at least two proven methods of vascular insufficiency was not performed in the studies.	Hukuda and Wilson, 1972; Uchida et al., 2003; Cheung et al., 2009; Kubota et al., 2011; Prange et al., 2012
Evidence of myelin destruction or “demyelination” seen in histology	Poor Unclear whether this reflects axonal degeneration or primary demyelination. Multiple non-specific stainings were used. Quantification not performed	Hukuda and Wilson, 1972; Al-mefty et al., 1993; Baba et al., 1996; Kanchiku et al., 2001; Lu et al., 2001; Ozawa et al., 2004; Xu et al., 2006; Yu et al., 2009; Klironomos et al., 2011; Kadota et al., 2012; Prange et al., 2012; Karadimas et al., 2013a, 2015; Long et al., 2014; Jiang et al., 2016
“Axon degeneration” or “injury” using IH, DTI, histology“Reduction of axon regeneration and number of normal axons” using IH (RT-97 and SMI-31) and DTI	Poor Most studies did not cross validate findings and did not perform quantification	Kanchiku et al., 2001; Yamaura et al., 2002; Zhao et al., 2005; Hu et al., 2011; Kadota et al., 2012; Long et al., 2013; Karadimas et al., 2015; Dhillon et al., 2016; Jiang et al., 2016
**Mechanisms of injury**		
Increased apoptosis	Moderate Three studies cross validated findings with a second approach and two makers and provided strong evidence. Five studies used two approaches but only one maker. Five studies used a single approach and one marker and provided poor evidence for apoptosis.	Gooding et al., 1975; Baba et al., 1996; Liu et al., 2000; Zhao et al., 2005; Takenouchi et al., 2008; Yu et al., 2009, 2011; Kurokawa et al., 2011; Dhillon et al., 2016; Yoshizumi et al., 2016
Increased inflammation Upregulation of inflammatory pathways/cytokines	Poor Single approach to confirm findings with one marker. Moderate – low There was no cross validation of results looking at e.g., BDNF/NT3. Functional tests in animals where inflammatory pathways were being investigated were lacking.	Baba et al., 1996; Yato et al., 1997; Yamaura et al., 2002; Ozawa et al., 2004; Penny et al., 2007; Uchida et al., 2008; Kubota et al., 2011; Kurokawa et al., 2011; Yu et al., 2011; Hirai et al., 2013; Moon et al., 2014; Dhillon et al., 2016

## Discussion

### Pathophysiology of DCM

**Figure 2:**
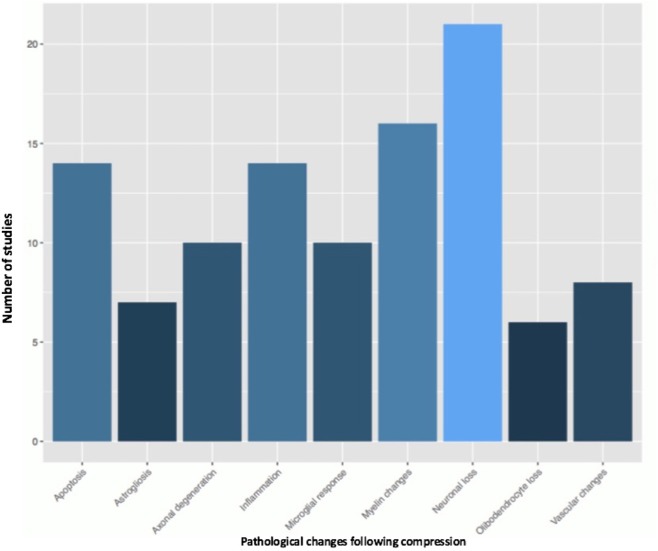
Pathological changes found in the chronically compressed cervical spinal cord.

**Figure 3:**
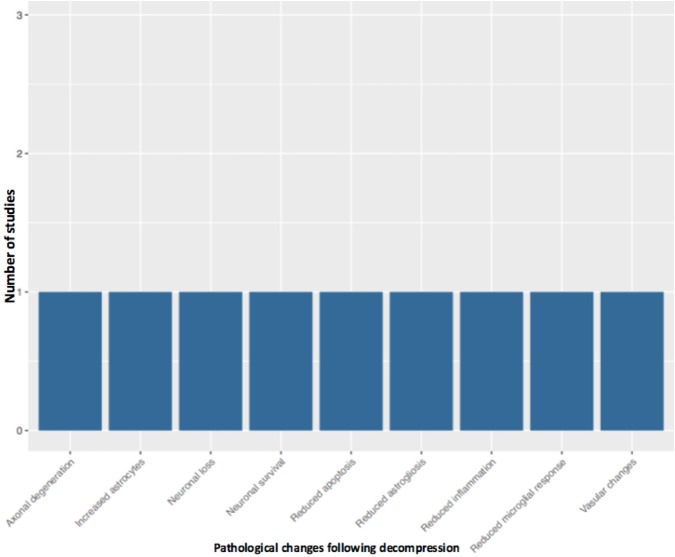
Pathological changes in the decompressed spinal cord.

**Figure 4:**
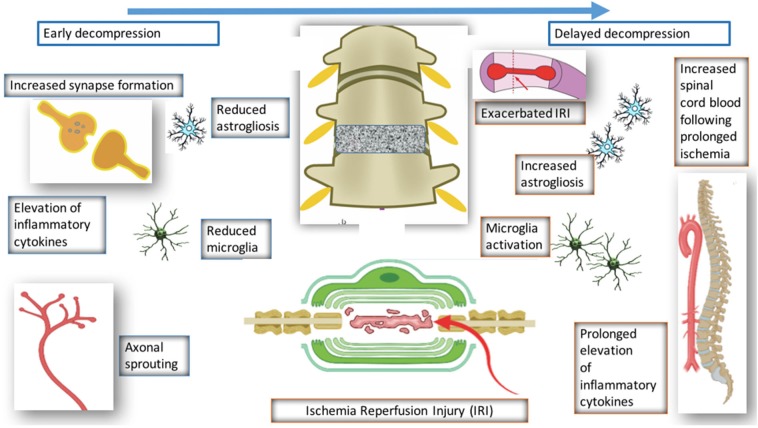
Pathological changes following decompression.

Fifty-one studies addressed the pathophysiology of DCM (Table 2; Tables S1, S2; Figure 2) or physiological changes following decompression (Table 2; Table S3; Figures 3, 4). The most common mechanisms involved in the pathophysiology of compression include cell apoptosis, vascular changes, inflammatory responses, axon degeneration and myelin changes (Figure 2). Fourteen papers studied the extrinsic apoptosis pathway. Fourteen papers studied the role of inflammation, of which 10 papers explicitly assessed the microglial response following chronic cord compression. Eight groups studied the vascular changes seen following chronic compression of the spinal cord. Sixteen assessed myelin changes after chronic compression injury. Ten studies investigated axon degeneration in DCM pathogenesis. Twenty-one papers investigated the compressive effects on neurons. Seven papers investigated presence of astrogliosis in DCM. Six studies demonstrated loss of oligodendrocytes following chronic cord compression. Four papers investigated the physiological changes following decompressive surgery (Figure 3).

#### Structural Changes

Structural damage in pre-clinical DCM models has not been fully investigated. A number of histological studies describe structural changes in the compressed spinal cord, which include vacuolar degeneration (Gooding et al., 1975; Al-mefty et al., 1993; Klironomos et al., 2011; Long et al., 2014; Jiang et al., 2016), interstitial oedema (Al-mefty et al., 1993), mitochondrial oedema (Long et al., 2013), cytoplasmic reduction (Aung et al., 2013) and cavity formation. Novel imaging techniques such as diffusion tensor imaging (DTI) with evidence of decreased diffusivity and fractional anisotropy at the compressed site suggesting reduced microstructural integrity (Yu et al., 2011).

#### Cellular Changes

##### Neuron loss

It has been well-documented in post-mortem studies that spinal cord compression leads to neuronal and axonal degeneration in the anterior horn (Yu et al., 2011), with evidence of loss of interneurons (Ogino et al., 1983) and lower motor (Ito et al., 1996). Animal studies also demonstrate loss of neurons in the gray matter of the ventral horns (Kanchiku et al., 2001; Kim et al., 2004; Klironomos et al., 2011; Dhillon et al., 2016; Jiang et al., 2016; Yoshizumi et al., 2016). Compression occupation rate is thought to be an important factor in neuronal survival, with a level >30% reducing levels of cholinergic neurons (Yu et al., 2011) and large motor neurons (Yato et al., 1997). In contrast to acute spinal cord injury, DCM patients often have preserved respiratory function. In one elegant study, it was shown that an excitatory neural network in the mid-cervical region is responsible for maintenance of breathing in DCM providing insight into how excitatory interneurons could be used to modulate respiratory function in acute spinal cord injury (Satkunendrarajah et al., 2018). Neuron loss can occur due to a number of factors, including apoptosis, inflammation or ischemia. Neuron loss appears to be mediated by various innate immune responses with evidence of increased levels of pro-inflammatory cytokines and macrophages (Al-mefty et al., 1993). The role of the adaptive immune system, if any, has not been investigated in this model. Moreover, the exact interplay between the different modes of injury is also not fully understood.

##### Axon degeneration

Chronic cord progression leads to axonal destruction at the site of compression (Kubota et al., 2011) and at sites cranial and caudal to the compression site (Prange et al., 2012). Neuroaxonal changes in compressed cords can be studied by immunoreactivity (IR) for amyloid precursor protein (APP) and phosphorylated neurofilaments (SMI312, SMI31, SMI32). Chronic compression is associated with increased APP at the compressed site (Dhillon et al., 2016). APP is transported by axoplasmic flow and accumulates when there are cytoskeletal defects (Dhillon et al., 2016). However, the mechanism underlying this is not well-known.

There is evidence of both retrograde degeneration and Wallerian degeneration of axons (Karadimas et al., 2013b) and this leads to irreversible damage to structural components of axons such as neurofilaments (Wang et al., 2012). Phosphorylated neurofilaments such as SMI-31 are found in normal axons and can therefore be used as a marker of healthy axons. In chronically compressed cords, there is evidence of a reduction in SMI-31–positive fibers (Takano et al., 2013). There is evidence of widespread axonal damage in the lateral and posterior funiculi (Kanchiku et al., 2001) and slight axonal damage in the anterior funiculi of compressed cords in rabbits (Ozawa et al., 2004). Histological analysis reveals evidence of Wallerian degeneration in the lateral funiculi at sites caudal to the compressed region in sheep (Penny et al., 2007). Early studies in dogs show slight degrees of demyelination in the ventral funiculi and the dorsal part of the lateral funiculi (Harkey et al., 1995). To further assess neuronal damage, one study investigated the serotonergic (5-HT) axons of the descending raphespinal tract in the spinal cord and found that compression was associated with a significant loss of 5HT- positive axons at the center of compression (Dhillon et al., 2016).

##### Oligodendrocyte loss and demyelination

Oligodendrocytes are supporting cells of the central nervous system (CNS) involved in myelination of axons. **Primary** loss of oligodendrocytes therefore leads to denuded axons, which display impaired axon conductance (Franklin and Ffrench-Constant, 2008). Six studies demonstrated apoptosis of oligodendrocytes in chronic compression models (Liu et al., 2000; Zhao et al., 2005; Takenouchi et al., 2008; Inukai et al., 2009; Uchida et al., 2012; Moon et al., 2014). This was predominantly found in the twy (tiptoe walking—Yoshimura) mouse model, an autosomal recessive mutant model which leads to calcification of the C1-C2 region and compression of the spinal cord. However, these studies typically only confirmed oligodendrocyte loss using one technique with little evidence of quantitative analysis. Many studies used techniques such as Luxol Fast Blue (LFB) staining to demonstrate myelin sheath changes, however electron microscopy, considered the gold standard in demonstrating evidence of nude axons and thereby demyelination was not performed (Franklin and Ffrench-Constant, 2008). A number of studies cross-validated their data using SEP by measuring the amplitude giving useful information about axon density and latency, which is prolonged following demyelination (Mallik and Weir, 2005), however these should be performed in addition to electron microscopy.

Axonal degeneration leads to **secondary demyelination**, with consequential loss of oligodendrocytes. More rigorous attempts are mandated in order to differentiate between primary and secondary demyelination in DCM models.

##### Astrogliosis

Astrogliosis is a reactive process that occurs in response to damage to nearby structures such as neurons. It can be typically demonstrated using immunohistochemistry and the presence of glial fibrillary acidic protein (GFAP). Chronically compressed cords appear to be associated with increased expression of GFAP in a number of different models (Hukuda and Wilson, 1972; Harkey et al., 1995; Yu et al., 2009; Moon et al., 2014). However, one study found evidence of astrogliosis not in the compressed region, but in areas proximal and distal to the maximal compressed site (Long et al., 2014). This may occur to protect intact regions of the cord from further damage and separate the damaged region from normal regions. The exact timing when the studies were performed following compression may also have an impact on the different results obtained. This is important to determine as prolonged astrogliosis may have detrimental effects. An understanding of molecular aberrations underlying astrogliosis has also not been investigated fully in these models. Further studies are required to quantify the loss of astrocytes with multiples markers and at different time points following compression.

#### Mechanisms of Injury

##### Apoptosis

Apoptosis is a form of programmed cell death found in both acute spinal cord injury and in chronically compressed spinal cords. It is possible that the mechanical stress associated with chronic cord compression directly leads to activation of pro-apoptotic factors, and this has been hypothesized in the case of traumatic brain injury (Ng and Lee, 2019). Alternatively, apoptosis may occur as a consequence of other destructive processes, such as inflammation or hypoxic damage. Apoptosis can be mediated by various cell signaling processes involving cell surface death receptors such as those from the tumor necrosis factor receptor (TNFR) family (Inukai et al., 2009; Uchida et al., 2012; Takano et al., 2014). Apoptosis can also be activated by intrinsic pathways initiated by mitochondrial damage and are characterized by the presence of apoptotic proteins such as Bcl-2 (Yu et al., 2011). Our analysis reveals an important role of the extrinsic pathway in apoptosis of cells in DCM. A number of studies have shown evidence of increased apoptosis mediated by the Fas pathway (Moon et al., 2014) activating caspase (Yu et al., 2011) which can be used as a therapeutic target. However, a time course analysis of apoptosis appears to be lacking in the current studies. Furthermore, other mediators of cellular apoptosis such as oxidants have also not been investigated.

##### Vascular changes

Changes in vascularity is commonly seen following acute spinal cord injuries (Figley et al., 2014) and appears to play a significant role in DCM. Microvascular swelling has been described following compression (Yamaguchi, 1980; Jiang et al., 2016), and proliferation of arterioles has also been described in one study (Al-mefty et al., 1993). Venous thickening and sinusoidal swelling has been described in three papers (Penny et al., 2007; Wang et al., 2012; Yamamoto et al., 2014). Vascular dysfunction was demonstrated in both the early studies performed in dogs and in more recent studies. Damage to the vasculature can lead to myelin damage (Gooding et al., 1975), glial fibrosis and necrosis (Ng and Lee, 2019). Early studies however were limited by small samples and limited quantitative analysis. Modern imaging techniques have attempted to visualize the exact vascular damage that occurs in DCM. Chronic cord compression can lead to damage to anterior spinal artery (ASA) (Long et al., 2014; Cheng et al., 2015) and ARA (Kurokawa et al., 2011). Vascular dysfunction appears to be associated with reduced blood flow to the spinal cord (Franklin and Ffrench-Constant, 2008; Kurokawa et al., 2011) and this can be demonstrated with various methods such as the microsphere technique which allows blood flow measurement using a fluorescence microsphere and avoids directly manipulating the spinal cord. However, injection of the microsphere in small animals can itself cause haemodynamic abnormalities (Prinzen and Bassingthwaighte, 2000). Other methods include the hydrogen clearance method, used by Al-mefty et al. (1993) and Harkey et al. (1995). This is an adaptation of the Fick principle where blood is linearly related to the local hydrogen concentration. Hydrogen clearance can be detected in any tissue where an electrode can be placed. Unlike microsphere techniques it allows repeated measurements of blood flow. However, the hydrogen clearance method often manipulates the spinal cord, which can affect the blood flow measurement. If there is a disruption of the gray matter/white matter interface after compression, this method cannot be used to determine blood flow in the two areas (Stalberg et al., 1998).

##### Hypoxia

Hypoxic changes in DCM have not been widely investigated. Tanabe et al. (2011), demonstrated increase in HIF-1α, a transcriptional regulator of oxygen homeostasis in the Twy/Twy model. However, their findings were not substantiated by investigation of other hypoxia induced factors, angiogenesis markers or the presence of reactive oxygen species. Furthermore, HIF-1α activates transcription of genes encoding various other factors including vascular endothelial growth factor (VEGF) (Ramakrishnan et al., 2014) and this needs to be investigated in relation to hypoxia in the DCM model.

##### Inflammation

A number of studies have shown evidence of increased inflammatory cells such as microglia in DCM (Moon et al., 2014), which increased with worsening cord compression (Hirai et al., 2013). Activated microglia in the form of the pro-inflammatory cells expressing markers such as TNF-α and CD86, are present in chronically compressed spinal cords (Ng and Lee, 2019). The role of the anti-inflammatory microglia is less clear with some studies showing no significant differences between control and compressed cords (Takano et al., 2013), whilst other studies showing these are present (Hirai et al., 2013) perhaps as a mechanism of recovery. Against, the time course when the activated microglia appear are not fully clear and this may help us determine the precise role of microglia activation patterns. Various other cytokines are also increased in compressed cords such as Th1 cytokines (Hirai et al., 2013) and increased TNFR1, CD95, and p75NTR due to increased chemokine signals (Takano et al., 2013). Inflammatory signaling pathways such as the NF-κB pathway is also upregulated in DCM (Karadimas et al., 2013a).

##### Changes in the extracellular matrix

The extracellular matrix (ECM) surrounding neurons in the form of perineuronal nets is composed of hyaluronan, proteoglycans and tenascin. Hyaluronan is released into the extracellular space and subsequently localizes around astrocytes, oligodendrocytes and neuron cell bodies (Eggli et al., 1992; Struve et al., 2005). The Hyaluronan tetrasaccharide (HA4), (hyaluronan degradation product), is known to have regenerative properties (Torigoe et al., 2011), and in a rat model of DCM appears to increase following 3 weeks of compression (Wang et al., 2012). This is associated with increased cellular inhibitor of apoptosis protein-2 (cIAP2) as well as a gradual reduction in apoptosis. There was also increased BDNF and VEGF expression at 4 weeks post-compression compared with the control group likely induced by astrocytes to aid in repair of neurons and oligodendrocytes. These results however have not been fully replicated in other studies, with some showing evidence of reduced BDNF levels in compressed cords (Uchida et al., 2003). Matrix metalloproteinases (MMP) are associated with dysfunction of the blood-brain barrier and increased levels of MMP in DCM has been described in one paper (Karadimas et al., 2013a).

#### Mechanisms of Recovery Following Decompression

Surgical decompression of the compressed spinal cord leads to partial recovery in patients and preclinical models of DCM. However, the mechanism underlying this recovery has seldom been investigated. Four studies have investigated the decompressed state of DCM (Harkey et al., 1995; Karadimas et al., 2015; Dhillon et al., 2016). Harkey et al. (1995) experimented on 12 dogs, which were compressed with an anterior screw device, followed by decompression of six dogs. The decompressed group demonstrated neurological improvement; however, there were no significant differences in SEP amplitudes and latencies, spinal cord blood flow, or MRI imaging characteristics between the compressed and decompressed groups. Histopathology was carried out on spinal cord tissue from the decompressed group, and this showed variable changes including necrosis and cavitation. Despite the use of higher order species, there were several limitations of the study such as the lack of quantitative data. Furthermore, majority of the data is descriptive and there no evidence of any systematic approaches to the study.

Dhillon et al. (2016) performed a study on 15 rats, which were randomly and equally allocated to control, compressed and decompressed groups. Evidence of axonal plasticity was observed following decompression. In the compressed group, increased levels of axon injury was observed using the marker APP, together with decreased numbers of serotonergic axons. Following decompression, there was increased GAP-43, which indicates increased axon growth rates, and increased 5HT+ axons and 5HT+/synaptophysin were also demonstrated, providing evidence for axonal sprouting.

Karadimas et al. (2015) demonstrated evidence of ischemia-reperfusion injury post decompression in a rat model, and suggested that this could be one of the reasons why some patients don't fully recover following decompression surgery. They demonstrated that decompression-induced ischemia-reperfusion injury however could be prevented by the administration of Riluzole and this can lead to better long term neurological outcomes compared to the group that received decompression surgery alone.

Vidal et al. (2017) investigated the consequences of delayed surgical management in DCM. Decompression is associated with various changes such as reduced microglia activation and reduced astrogliosis. However, when decompression is delayed, there is increased astrogliosis, microglia activation, and reduced spinal blood cord flow. Decompression itself is associated with ischemia reperfusion and elevation of inflammatory cytokines, however if decompression is delayed, this is prolonged and leads to worse neurological outcome. This highlights the importance of a therapeutic window in DCM and also the need to operate early with or without the use of any medical adjuncts.

#### Signaling Pathways and Therapeutic Strategies Explored for Promoting Recovery in DCM

Therapeutic signaling pathways which have been used to promote recovery in DCM include the brain derived neutrophic factor (BDNF) signaling pathway (Uchida et al., 2003; Xu et al., 2006; Hirai et al., 2013), Neurotrophin-3 (NT-3) pathway (Uchida et al., 1998, 2008; Hirai et al., 2013), Nuclear factor–[kappa] B (NF-[kappa]B) pathway (Karadimas et al., 2013a), Mitogen Activated Protein Kinase (MAPK) (Takenouchi et al., 2008) and phosphodiesterase (PDE)/cylic AMP pathway (Yamamoto et al., 2014). Other therapeutic agents used include prostaglandins (Kurokawa et al., 2011), granulocyte-colony stimulating factor (G-CSF) (Yoshizumi et al., 2016) and Riluzole (Karadimas et al., 2013a).

##### Brain-derived neurotrophic factor (BDNF)

BDNF is a member of the neurotrophin family and is important for survival of neurons (Bathina and Das, 2015). BDNF signaling is elicited after it binds to TrkB receptor. Chronic cord compression appears to reduce BDNF at the compressed site in the Twy/Twy mice model (Uchida et al., 2003). There is an increase at rostral and caudal sites suggesting that in the compressed model there is an attempt to promote regeneration. However other studies have demonstrated that with an increase in duration of compression, there is increased BDNF intensity (Hirai et al., 2013). Xu et al. (2006) showed that administration of BDNF can increase the number of neurons at the compressed spinal cord. It appears from the above studies that the response of BDNF in compressed cords is not fully characterized. A full understanding of its response at different times during compression and decompression is warranted.

##### Neurotrophin

Neurotrophin (NT-3) are a family of growth factors involved in neuron survival and axonal regeneration (Lu et al., 2001). Compression of the spinal cord appears to downregulate the levels of NT-3 (Uchida et al., 1998, 2003) at the epicenter and also downregulates the levels of its receptor, trKC (Uchida et al., 2003). However, there is upregulation rostral to the compressed site (Uchida et al., 1998, 2003) and caudal to the compressed site (Uchida et al., 2003). Targeted retrograde gene delivery of NT-3 has been shown to reduces loss of neurons seen in compressed cords and improves their morphology (Takenouchi et al., 2008). Targeted retrograde gene delivery of NT-3 therefore may be used to promote neuronal survival in compressed spinal cords.

##### Mitogen-activated protein kinase (MAPK)

The MAPK signaling pathway is an intracellular signaling system that controls many basic cellular functions, including apoptosis (Chang and Karin, 2001), which induces optimal stress responses. It encompasses a core signaling module, which includes MAPK kinase kinase (MAPKKK), MAPK kinase, and MAPK (Chang and Karin, 2001). One of the MAPKKKs is apoptosis signal related kinase 1 (ASK1), which is activated by reactive oxygen species and tumor necrosis factor (TNF)-[alpha] (Long et al., 2013). Other stress-activated MAPK pathways include JNK and p38. All three pathways were upregulated in chronically compressed cords and located in apoptotic cells (Takenouchi et al., 2008). Similar dysregulation of MAPK has been found in mechanically-induced apoptosis of chondrocytes (Kong et al., 2013) supporting the notion that inhibitors of ASK1-JNK/-p38-caspase pathway mediated apoptosis may be a therapeutic target in DCM.

##### Phosphodiesterase (PDE)/cyclic AMP

The phosphodiesterase (PDE)/cyclic AMP pathway has been investigated in one study. Phosphodiesterase are enzymes that degrade the phosphodiester bond in the secondary messengers, cAMP and cGMP. PDE inhibitors inhibit degradation of these messengers and thereby enhance their effects, in part via modulation of MAPK. The PDE3 inhibitor Cilastazol has been shown to inhibit platelet aggregation and acts as a vasodilator by inhibiting activation of myosin light chain kinase, which contracts smooth muscle cells. Cilastazol has been shown to improve neurological function, reduce neuron loss and apoptosis in a rat model of DCM (Yamamoto et al., 2014).

##### Nuclear factor–[kappa] B (NF-[kappa]B)

Nuclear factor–[kappa] B (NF-[kappa]B) are transcriptional factors, composed of the Rel family of proteins including RelA (p65), NF-[kappa]B1 (p50), and NF-[kappa]B2 (p52) (Christian et al., 2016). The most common form is the NF-[kappa]B1-RelA that contains the p65 and p50 proteins. Resting NF-Kb in the cytoplasm becomes activated after phosphorylation of its inhibitory component, followed by ubiquitination, and degradation. The NF-[kappa]B then translocate to the nucleus and binds to target genes involved in various functions (Christian et al., 2016). The role of NF-KB has been investigated in one study in rabbits (Karadimas et al., 2013a). Chronic compression of the spinal cord leads to increased levels of nuclear and cytoplasm p50 and p60. However, the exact effects of activated NF-Kb in DCM is not known. The role of these transcriptional factors in the recovery of DCM has not been investigated.

##### Prostaglandins

Prostaglandins are lipid compounds found in most tissues with diverse functions. A prostaglandin PGE1 derivative Limaprost has been shown to reduce neuron loss seen in the rat DCM model (Kurokawa et al., 2011). Limaprost is an anti-platelet agent and a vasodilator and it is thus possible it played a vasodilatory role in the DCM model. However, how the drug prevented neuron loss was not clear (Kurokawa et al., 2011).

##### Granulocyte colony stimulating factor (G-CSF)

Granulocyte colony stimulating factor (G-CSF) is a 19.6-kD glycoprotein that has neuroprotective effects in experimental spinal cord injury. It can induce proliferation of oligodendrocyte precursors, inhibit apoptotic cell death, and suppress inflammatory cytokines (Kadota et al., 2012). G-CSF has been shown in one study to reduce the decline in motor function and loss of motor neurons in a rat model of DCM (Yoshizumi et al., 2016). G-CSF was also shown to restore motor function with the effectiveness observed at approximately 8 weeks. The authors suggested that surgical decompression may be considered during that period to enhance the benefits of the drug.

##### Riluzole

Riluzole is a sodium glutamate antagonist, which appears to be neuroprotective in acute and chronic injuries of the CNS. Riluzole decreases NMDA receptor phosphorylation in astrocytes and can reduce microglia activation in the chronically compressed spinal cord (Moon et al., 2014). In the decompressed spinal cord, there is evidence for ischemia-reperfusion injury, which may be responsible for initial post-operative neurological decline after decompression surgery. This process can be attenuated using Riluzole, which appears to reduce oxidative DNA damage in the spinal cord (Karadimas et al., 2015).

##### Autophagy

Autophagy is a mechanism controlling the turnover of organelles and proteins in cells. Autophagy can act to prevent apoptosis and therefore could be exploited therapeutically. One study investigated the neuroprotective role of autophagy in DCM. Tanabe et al. (2011) demonstrated increased expression of autophagy markers in compressed spinal cords and this was associated with a reduction in neuronal apoptosis.

## Conclusions and Recommendations

In this systematic review we were able to delineate some of the core pathophysiological processes that have been described in DCM. Our main findings however demonstrate that this condition is not well-studied, and numerous key questions remain regarding the pathobiology of this condition thereby hindering progress in developing therapeutics to improve outcomes. We have highlighted the key processes involved in this condition such as axonal degeneration, apoptosis of cells, and vascular dysfunction. However, how these process lead to cellular and molecular changes in the condition has not been well-studied. We have demonstrated the lack of understanding of the changes that occur following decompression. In addition to early surgery, there is a therapeutic window in which outcomes can be improved. Further studies investigating the mechanisms of injury and recovery in the decompressed spinal cord and how these can be modulated for patient benefit is warranted.

## Recommendations

We recommend the following based on the results of our systematic review

1. Define standardized protocols for pre-clinical DCM models, including outcome measures that are relevant to the clinical setting.
2. Delineate the intrinsic and extrinsic mechanisms responsible for axonal degeneration and cell death in DCM, and in particular how mechanical stress translates into cellular injury.
3. In human patients the extent of cord compression correlates poorly with neurological function. It is therefore important to determine mechanisms, genes and pathways that are able to modulate the neurological decline caused by chronic spinal cord compression.
4. Investigate whether intra-cellular repair mechanisms such as autophagy can prevent neuronal and axonal loss, halt disease progression or promote outcomes in DCM.
5. Investigate the role of key cellular elements of the CNS in the decline of neurology caused by cord compression and the recovery following decompression, including the role of oligodendrocytes, astrocytes, microglia, pericytes, etc.
6. Investigate the role of inflammation, and in particular the innate immune response, cytokine profiles and signaling during compression and following decompression.

## Data Availability Statement

All datasets generated for this study are included in the article/supplementary material.

## Author Contributions

FA, XY, and MK: manuscript design, data collection, analysis, drafting of manuscript, editing of manuscript. XQ and SY: analysis, drafting of manuscript, editing of manuscript. PN and YS: drafting of manuscript, editing of manuscript.

## Conflict of Interest

The authors declare that the research was conducted in the absence of any commercial or financial relationships that could be construed as a potential conflict of interest.
