# Corrigendum: The Pathophysiology of Degenerative Cervical Myelopathy and the Physiology of Recovery Following Decompression

**DOI:** 10.3389/fnins.2020.00628

**Published:** 2020-07-17

**Authors:** Farhana Akter, Xinming Yu, Xingping Qin, Shun Yao, Parisa Nikrouz, Yasir Ahmed Syed, Mark Kotter

**Affiliations:** ^1^Department of Clinical Neuroscience, University of Cambridge, Cambridge, United Kingdom; ^2^Faculty of Arts and Sciences, Harvard University, Cambridge, MA, United States; ^3^Massachusetts General Hospital Cancer Center, Harvard Medical School, Boston, MA, United States; ^4^Department of Neurosurgery, Brigham and Women's Hospital, Harvard Medical School, Boston, MA, United States; ^5^Maidstone and Tunbridge Wells Trust, Maidstone, United Kingdom; ^6^Neuroscience and Mental Health Research Institute (NMHRI), Cathays, United Kingdom; ^7^School of Bioscience, Cardiff University, The Sir Martin Evans Building, Cardiff, United Kingdom

**Keywords:** neuronal loss, apoptosis, pathogenesis, spine, degeneration

In the published article, an author name was incorrect as Yasir Syed. The correct name is Yasir Ahmed Syed.

In the original article, we neglected to include the funder Cambridge NIHR Brain Injury MedTech Cooperative, NIHR Clinician Scientist Award CS-2015-15-023 to Mark Kotter. The updated Funding statement appears below.

## Funding

We gratefully acknowledge support by the Cambridge NIHR Brain Injury MedTech Cooperative. MK was funded by a NIHR Clinician Scientist Award CS-2015-15-023.

## Disclaimer

This report is independent research arising from a Clinician Scientist Award, CS-2015-15-023, supported by the National Institute for Health Research. The views expressed in this publication are those of the authors and not necessarily those of the NHS, the National Institute for Health Research or the Department of Health.

The authors apologize for these errors and state that this does not change the scientific conclusions of the article in any way. The original article has been updated.

