# Supplementary material for: The Pathophysiology of Degenerative Cervical Myelopathy and the Physiology of Recovery Following Decompression
Source: Front Neurosci. 2020 Apr 30;14:138. doi: 10.3389/fnins.2020.00138 (PMC7203415; doi:10.3389/fnins.2020.00138)
Supplement: Supplementary file 1 [file Table_1.docx]

**Table 1: Pathophysiology of DCM**

CCC (chronic cord compression); dCCC (decompression of the chronically compressed group); IH (immunohistochemistry); WM (white matter); GM (grey matter); anterior horn (AH); posterior horn (PH); AC (anterior column); PC (posterior column); C (cervical spine)

| **Authors** | **Methods** | **Results** | **Key Findings (KF)**  **Limitations (L)** |
| --- | --- | --- | --- |
| **Hukuda et al, 1972 (^34^)** | Animals: Dogs  Control= 5  Compression = 8 (Total of 28 in experiment)  Screw compression at cervical spine level 5 (C5)  Direction of compression: Anterior  Duration of compression: 6 months and longer for additional experiments | Maximum tolerable compression (MTC):  Screws tightened in 1mm increments until dogs develops limb weakness then removed  Neurology: (points given for weakness in forelegs, hindlegs and accentuated knee jerk- higher scores represent worse abnormality)  28 dogs (+5 dogs as controls) 15/28 kept for further procedures  5/28 not analysed  18/28 had compression <45%- 14 of these had no neurological deficit, 4 had mild deficit  8/28 had neurological deficit at 45% compression  Microangiography: there was a contrast filling defect, greatest at the compressed C-6 segment,  Histology: Haemotoxylin and Eosin (H&E), Weil’s, KB, Nissl’s, Marchi  Flat white matter (WM)/grey matter (GM), neurons shrunken, demyelination in ventral WM  Chronic vascular insufficiency I (CVI):  12 dogs  1^st^ operation= anterior spinal artery (ASA), bilateral vertebral arteries (VA) and bilateral radicular arteries were ligated at C1.  2^nd^ operation= Ventral radicular arteries doubly ligated at C3, and divided.  Sham CVI Operation. In five dogs the two operations were performed but no arteries were interrupted. These dogs served as controls for the acute bleeding test.  Moderate weakness in 3/12 animals, transient weakness in 9/12 animals.  CVI- neurological score= 1.4  Histology:  Demyelination (DM) in ventral WM peripheral region  Slight DM in fasciculus cuneatus in 2 dogs and slight DM in dorsal spinocerebellar tract in 4 dogs  Chronic vascular insufficiency II (CVII):  The cervical vertebral arteries were obliterated on both sides with solid polyethylene tubes to eliminate possibility of reversed blood flow from the brain.  Neurological score = 0.8  MTC then CVI:  15 dogs with MTC used  -4 dogs- CVI established with 2 stage procedures  -11 dogs- CVI-II was established in one-stage operations.  Neurology: Moderate weakness in all dogs  Neurological score= 1.0  Microangiography: reported as not done as numbers too small for statistics  Histology: Same as MTC/ CVI  MTC+CVII:  Marked neurological weakness in all dogs  Neurological score= 3.2  Microangiography: there was a contrast-filling defect, greatest at the compressed Cervical spine level 6 segment.  Histology:  Same as MTC/ CVI. Also, 6/11 had in addition necrosis, reduced nerve cells, glial fibrosis in GM, demyelination, reduced nerve cells in WM.  Quantitative evaluations of histological abnormalities showed that the C-6 segment and the C-5 segment had higher points representing extent of abnormality.  Acute Bleeding Test. This was done before and after the CVI-I operation in eight dogs, before and after the CVI-II operation in five dogs, and before and after the CVI sham operation in five dogs.  The dogs were bled until they were unable to stand even when helped; the point at which the dogs began to sit down repeatedly in spite of efforts to keep them standing was regarded as the "onset of weakness.  The onset of weakness was soonest in the group with the worst neurological scores (MTC and CVII). | KF:  The effects of compression and vascular insufficiency are additive, leading to worse neurological dysfunction than compression or vascular insufficiency alone.  L: Histology stainings were not specified  No statistical tests performed  Highly invasive/unethical procedure  Large numbers lost to follow up |
| **Gooding et al, 1975 (^41^)** | Animals: Dogs  Control= 4  Compression= 10  CVI= 6  Methods: Screw between C4-C5 vertebral bodies  Direction of compression: anterior  Duration of compression: 2 hours | Maximum tolerable compression (MTC):  10 dogs- screws tightened in 1mm increments until dogs develops limb weakness then removed  Neurological outcome: (clinical examination, no scoring method)  6/10 dogs🡪 mild-mod tetraparesis  4/10🡪 mild paraparesis  Histology:  Marchi method, H&E  No comment regarding pathology  Chronic vascular insufficiency I (CVI):  6 dogs- CVI induced with bilateral lateral spinal artery (LSA) ligation at C3,followed by ASA ligation at C1, vertebral artery (VA) ligation at proximal to C6  Neurological outcome  3/6🡪 minimal tetraparesis  Histology: demyelination in fasciculus cuneatus, Anterior column (AC)/lateral column (LC) of WM  MTC & CVI:  After complete neurological recovery (range 3-13 days), 10 dogs underwent MTC then CVI  4 dogs served as controls  Neurological outcome  10/10🡪 neurological deficit  5/10 🡪 tetraparesis  Histology: more pronounced than CVI, Demyelination widespread in WM.  4/10 Demyelination of post corticospinal tract  CVI then later MTC:  In 3/6 dogs with CVI, MTC performed later to determine if reversal of sequence of procedures influence outcome  Neurological outcome  3/3 were normal after CVI but after MTC developed ataxia, paraparesis  Histology: Degeneration of junctional zone around anterior horn (AH)  2/3 Demyelination of post corticospinal tract | KF:  Local deformation and ischemia leads to neurological dysfunction seen in DCM  L: No statistical testing performed |
| **Yamaguchi et al, 1980 (^12^)** | Animals: Rabbits  Control= 10  Compression= 39  Group A- compression ratio (CR)= 10% n=10  Group B- CR=11-30%- n=9  Group C- CR= 31-50%- n=15 (6 developed myelopathy)  Group D – CR= 51%- n= 5- all animals here developed acute cord injury  Methods: Screw through C4-5 disc space (anterior)  Direction of compression: anterior  Duration of compression: F/u for 1,3,6 months | Neurology  Group A/B: No deficit  Group C:  6/15 developed myelopathy, 9/15 had no deficit  Day 1-7: Normal neurological function  Day 14- 42: Unstable, spastic gait, motor weakness in limbs,  hyper-reflexia  Group D: Signs of acute spinal cord injury.  EM:  CCC:  Pyknotic and irregular nucleus membrane, oedematous mitochondria, degeneration of endoplasmic reticulum and synaptic vesicles, myelin sheath attenuation, dilatation of the periaxonal spaces especially in the WM.  In the GM, capillary narrowing and oedema, mild swelling of astrocytic processes.  Myelopathy group- oedematous change and axonal regeneration in affected nerve fibres stronger in caudal region than rostral. | KF:  Evidence of microvasculature oedema and axonal regeneration in myelopathy.  L: Descriptive study only. |
| **Shinomiya et al, 1992 (^69^)** | Animals: Cats  Control= 0  Compression= 6  Methods: Screw compression at C4 vertebral body (n= 3) and multiple screw compression at C4, C5, C6 vertebral body (n= 3)  Direction of compression: anterior  Duration of compression: 6 months | Histology: H&E, Kleihauer–Betke (KB)  Single compression:  Slight change in anterior horn (AH)/(PH), mild demyelination in anterior column (AC)/posterior column (PC)  Multiple compression:  Severe GM damage, reduced cells, cavity formation  Demyelination in AC/PC  Segmental evoked potentials (SEP)  - Amplitude in N2 wave  -Conduction velocity (CV)  Single compression:  Moderate reduction in segmental SEP  Slower CV  Multiple compression:  Severe reduction in segmental SEP  Slower CV  Segmental SEP reflected histological changes in GM  (CV from AC depicted demyelination in AC. CV from PC depicted demyelination in PC) | KF:  Multiple compression levels leads to severe damage to GM and cells/myelin and reduction in somatosensory function.  L: No controls |
| Al-Mefty et al, 1993 (^8^) | Animals: Dogs  Controls= 4 (2/4 controls developed myelopathy)  Compression= 14 (12/14 dogs developed myelopathy)  Methods: Teflon screw C5 anteriorly to cause 30% compression  Direction of compression: anterior and posterior  Duration of compression: Up to 18 months | Histology: H&E, LFB, Modification of Weil stein (for myelinated axons), GFAP fluorescence tag for glial response  Loss of large motoneuron in grey matter (GM) & anterior horn (AH) necrosis, cavitation at junction of dorsal and anterior horns and site of greatest compression.  Magnetic resonance imaging (MRI):  5/14 developed lateral compression 9/14 developed central compression  Compression= average 29% (10-50%)  “Snake eyes’ (indicating myelomalacia of the central GM), seen in 14/14 compressed dogs and 3/4 controls.  Microangiography (analysed in 13/18)  Anterior spinal artery (ASA) perfused in 13/13  Radicular artery bilaterally perfused in 9/13, 3/13 decreased on 1 side, 1/13 decreased bilaterally  8/13 increased number of medullary vessels in grey matter (GM)  - No comparison of control vs. CCC provided  Spinal cord blood flow (SCBF (ml/min/10g)  - using hydrogen clearance method  Immediate increase following surgery (12.5) vs. pre-surgery (9) (p<0.05), final result= 10. Did not compare to controls.  SEP  Stated there is a prolonged N1 latency in CCC- but no values given  There was progressive deterioration of SEP with increased compression. | KF:  Progressive compression leads to reduced blood flow, deterioration in SEPs and neuronal loss, which are greatest at the site of maximal compression  L: No quantification, no cross validation of findings. 50% of controls also developed |
| Baba et al, 1996 (^70^) | Animals: Twy/Twy mice  Controls= 5 Compression= 50 (33/50 developed myelopathy)  Methods: Twy/Twy mice developed calcified deposits resulting in variable spinal cord compression at the level of C2-C3  Direction of compression: Posteriorly and posterolaterally  Duration of compression: 4-8 months | Histology: Nissl  Transverse remnant area of the spinal cord [TRAS (%)] = (Transverse area of SC with maximum compression at C1 in Twy/Twy mouse: corresponding area in control)  -There was correlation between TRAS i.e. maximum compression and number of motoneurons on compressed side (p<0.05) - On both sides, motoneuron numbers significantly decreased at site of compression (P<0.05) - On both sides, motoneuron numbers increased rostral to site of compression (P<0.05) - Contralateral to side of compression, there was a significant decrease in motoneuron numbers immediately rostral to the site of compression, in animals with TRAS of 70% or more, compared to controls (p<0.05) | KF:  Compression leads to neuronal loss at site of compression. There are increased number of neurons rostral to site of compression.  L: Study only included morphological changes in response to compression. No cross validation of findings.  Function of animals not assessed alongside morphology. |
| Yato et al, 1997 (^71^) | Animals: Twy/Twy mouse  Control= 12  Compression= 30  (anterior= 8, posterior=6, lateral=16)  Methods: Twy/Twy mice developed calcified deposits resulting in variable spinal cord compression at the level of C2-C3  Direction of compression: Lateral  Duration of compression: 24 weeks | Histology: H&E  Control: No change  CCC: Lateral- AH cell atrophy, ipsilateral cell loss; no difference between both halves in anterior/posterior compression.  Occupation rate (OR)  OR of 20%- no cell loss  OR of 20-30% - mod cell loss and atrophy  OR of >30%- all animals had cell loss and atrophy  Immunohistochemistry: Anti- Choline acetyltransferase (ChAT)  Controls: Neurons present in AH of  ChAT immunofluorescence intensity in AH showed a linear negative correlation with occupation rates P<0.0001)  ChAT immunofluorescence intensity at OR>20% lower than OR<20% p<0.05  ChAT immunofluorescence intensity lower in compressed side vs. contralateral p<0.05 | KF:  Occupational rates of at least 20% leads to functional changes in spinal motoneurons of cords, which were compressed laterally.  L: No functional tests |
| Uchida et al, 1998 (^59^) | Animals: Twy/Twy mice  Controls= 18  Compression= 70  Methods: Twy/Twy mice developed calcified deposits resulting in variable spinal cord compression at the level of C2-C3  Direction of compression: posterolateral  Duration of compression: 20 weeks | Transverse remnant area of the spinal cord [TRAS (%)] : 44.4  Immunohistochemistry (IH):  Wheat germ agglutinin-horseradish  peroxidase (WGA-HRP)-labelled accessory motoneurons  Neurotrophins: brain-derived neurotropic factor (BDNF) and neurotrophin (NT)-3  CCC: No of motoneurons in compressed site reduced vs. controls p<0.05  WGA-HRP-la- belled motoneurons in compressed site reduced vs. control p<0.01  Dendritic length (um) rostral to C1 (577) and rostral to c1-c2 (523.8) vs. corresponding controls (p<0.05). Decrease in length at compressed site (242.6) vs. control but not significant (ns)  Neuronal soma  -Small but insignificant decrease in the somal area at the site of compression (324um2)  -Enlargement of the neuron soma Rostral to c1 (574um2) and rostral to c2-c3 (565um3) vs. corresponding control p<0.05  BDNF  Control: immunoreactivity localised in GM  CCC: BDNF immunoreactivity increased with increased TRAS rostral to compressed site  NT3  NT-3 immunoreactivity tended to diminish at compressed site with increased duration of compression  In severe compression- increased astroglial like cells in WM with BDNF and increased NT3 reactivity in axons of AC seen rostrally | KF:  The area of neuronal soma and  total length of dendrites of  motoneurons  decreased significantly with decrement in motoneuron population, relative to the control.  There was increased functional activity of anterior horn cells at levels rostral to the site of compression and there was also enhanced BDNF and NT-3 immunoreactivities rostral to site of compression.  L: No cross validation or quantification of BDNF/NT3. |
| Kanchiku et al, 2001 (^19^) | Animal: 13 weeks old rabbit  Control= 5  Compression= 8  Methods: Compression of spinal cord with screw through the anterior C5 vertebral body followed by insertion of a plastic plate under the vertebral arch (posterior) of C5 two weeks later  Direction of compression: Anterior - posterior  Duration of compression: 10 months | Neurology: Modified Tarlov Score (MTS)  0 No voluntary movements  1 perceptible movement  at joints, hindlimbs follow  2 Good movement at joints but unable to stand up  3 Can stand up and walk, but unable to start running quickly  4 Normal  Modified Tarlov Score:  Control = 4  CCC= 3 (n=7), 4 (n=1)  Histology:  Neurofilament staining, KB stain, Verhoeff-Van Gieson elastic staining (EVG) stain  Histology: CCC  Neurofilament staining: axon degeneration, spongy degeneration, demyelination of the lateral funiculi and posterior funiculi.  KB stain: Ant horn flattening, loss of neurons in the anterior horn, gliosis  EVG stain: Venous wall thickening, lumen expansion in veins of the subarachnoid space + grey matter  SEP (brain SEP; Peripheral nerve SEP, spinal cord SEP)  Brain SEP  CCC (32), control (90), P <0.0001  Peripheral nerve SEP  CCC (42), control (105), p<0.01  Spinal cord SEP  CCC (50), control (1000), p<0.001  MRI  Compression ratio: ratio of AP diameter: transverse diameter  Cross section (mm2) in CCC (22) vs. control (23)  Compression ratio (%) in CCC (34) vs. control (52) p<0.01  Intramedullary high intensity areas at CCC are high. These areas were histologically correlated to necrotic changes and gliosis in the GM, with demyelination and axonal degeneration in the  WM. | KF:  Model used to demonstrate evidence of ability to produce compression.  All compressed animals had reduced amplitude of SEPs and histology revealed anterior horn cell necrosis with changes in the myelin and axons.  L:  No cross validation of findings |
| Uchida et al, 2002 (^72^) | Animals: Twy/Twy mice  Controls= 18 Compression= 54  (Aged: 8 weeks [n = 18]  14 weeks [n = 18]  20 weeks [n = 18])  Methods= Twy/Twy mice developed calcified deposits resulting in variable spinal cord compression at the level of C2-C3  Direction of compression:  Posteriorly  Duration of compression: 8, 14, 20 weeks | Histology: H&E, LFB, and toluidine blue 20 week old Twy/Twy mice: Spongy necrosis, myelin sheath separation, axonal swelling, axonal deformation, coarse nerve fibres, and expansion of nerve fibres with myelin sheath decay  Immunohistochemistry: anti-NF68 (neurofilament) and anti-GAP-43 antibodies  NF- protein required for cytoskeleton of mature axons  GAP-43(involved in regeneration and elongation of axons. It is an axonal transport membrane protein)  8weeks and 14weeks – weak immunostaining in WM for NF68, weak immunostaining in ventral horn and dorsal column for anti-GAP43  20weeks- strong immunostaining for GAP-43  Color image analysis: 20 week old Twy/Twy mice: GAP-43 immunoreactivity significantly higher in anterior and lateral funiculi, compared to posterior funiculi (p<0.05) | KF:  There is evidence of myelin sheath separation and axonal deformation in 20 week old mice. No such changes were noted in 8 week old mice.  Authors suggest the increased proportion of GAP-43 with increased magnitude of compression indicates possible involvement of GAP-43 in repair processes.  L: No functional assessments  No cross validation. No quantification. |
| Yamaura et al, 2002 (^73^) | Animals: Twy/Twy mice  Controls= 7  Compression= 7  Methods: Twy/Twy mice developed calcified deposits resulting in variable spinal cord compression at the level of C2-C3  Direction of compression: posterolateral  Duration of compression: 6 months | Histology:  H&E, KB, LFB  1month old mice: Spinal cord preserved  6month old mice: Compression occurred posterolaterally  GM: Reduced, flat, small neurons especially at posterior horn (PH)  WM: Myelin destruction, myelin debris in anterior (AC)/lateral (LC)/posterior column (PC).  Caudal to CCC: cavity formation, myelin debris in AC/PC (suggesting descending degeneration)  Rostral to CCC: cavity formation and myelin debris in PC (suggesting ascending degeneration)  Immunohistochemistry:  Caspase 3+ (mediator of apoptosis)  Caspase3+ cells present in GM/WM  Terminal deoxynucleotidyl transferase (TdT) dUTP Nick-End Labelling (TUNEL) staining (represents apoptotic cells)  Double staining: TUNEL + cells stained by oligodendrocyte (OLG) specific antibody  6month old mice  TUNEL + cells present in GM, WM, ant/post horn, ant/lat/post columns at compression site | KF:  Descending degeneration in the anterior and lateral columns and ascending  degeneration in the posterior column were observed.  The  distribution of oligodendrocytes with positive results  from TUNEL staining was similar to  that for degeneration of the long tracts, suggesting OLG apoptosis may contribute to long tract degeneration of the spinal cord.  L: No functional assessments  No cross validation.  No quantification. |
| Uchida et al, 2003 (^55^) | Animals: Twy/Twy mice  Controls= 24 Compression= 24 Methods: Twy/Twy mice developed calcified deposits resulting in variable spinal cord compression at the level of C2-C3  Direction of compression: Posteriorly  Duration of compression: 12-24 weeks | Immunohistochemistry: anti-BDNF, anti-NT-3, anti- Tropomyosin receptor kinase B (trkB) (BDNF receptor) anti- Tropomyosin receptor kinase (trkC) (NT3 receptor)–antibodies  Increased BDNF, NT-3, trkB, and trkC immunoreactivity rostral and caudal to compression site but decrease at epicenter of compression.  Densitometry:  Higher expression of BDNF, NT-3, trkB, and trkC at region rostral (p = 0.003) and caudal (p = 0.003) to the site of compression. Lower expression at the site of compression (p < 0.001) | KF:  In 24 week old mice with severe compression, there was lower expression of neurotrophins at site of maximal compression. In contrast, their expression was significantly higher at the rostral and caudal sites adjacent to the maximal compression site.  L: No functional tests, no histology |
| Kim et al, 2004 (^74^) | Animals: Male Wistar Rats 12-14w  Control= 12  Compression:  For 1 week N=6  For 3weeks N=6  For 9 weeks N=6  For 25 weeks N= 12  Methods: Polymer at C5-C6  Direction of compression: posterior  Duration: As above | Neurology  Voluntary exercise (VE)  (cumulative rotation of wheel in cage counted no of rotations per day)  VE  - No significant difference between controls vs. CCC  Forced locomotion capability (FLC)  Reduced in CCC after 17 weeks p<0.05  Histology: H&E stain  Cross-sectional area mm2 (CSA)  Bielschowsky silver staining (demyelination)  CSA  Reduced in 1,3,25weeks CCC groups vs. control p<0.05  Ventral horn distorted, reduced neurons, sinusoidal vein dilatation, grey matter cavitation  Reduced neurons at 3, 9, 25w vs. control (p<0.05, p<0.05, p<0.05)  Demyelination or pallor of myelin was not apparent in CCC.  Immunohistochemistry: anti-CD8 (microglia)  No increase in microglia in compressed cord segments. | KF:  This model reproduced the characteristic findings of human DCM. There was evidence of progressive motor disturbance and neuronal loss in the compressed group.  L: No cross validation of neuron numbers or myelin changes. |
| Ozawa et al, 2004 (^27^) | Animals: Rabbits  Control= 3  Compression= 12  Methods: Screw compression anterior C5 vertebral body  6- mild compression (MC) (2mm protrusion)  6- moderate compression (ModC) (3mm protrusion)  Direction of compression: anterior  Compression: 1 week | Compression ratio (CR)= ratio of the  area of the protruding screw in the spinal canal to the area of the spinal canal.  MC: 17; ModC: 31.8 P<0.0001  Compression area: ModC: reduced vs. control p<0.001  Histology:  H&E, KB (for axons and myelin and measurement of area)  KB staining  MC: Reduced neuron density, proliferation of inflammatory cells in AH ipsilaterally  ModC: Reduced neurons, increased cell proliferation in GM, slight demyelination at anterior funiculi  Immunohistochemistry:  Ricinus communis agglutinin (RCA-1) (reactive microglia), GFAP+ (astrocytes)  MC: proliferation of reactive microglia, enlarged cytoplasm in AH ipsilaterally ModC: significant proliferation of reactive microglia was observed in the GM ipsilaterally  GFAP+ astrocyte density- Increased ipsilaterally vs. control In AH (p<0.001)-, DH (P<0.01), Anterior funiculi (p<0.001),  Lateral funiculi (P<0.001- but no difference with contralateral side) and dorsal funiculi (P<0.001- but no difference with contralateral side)  As compression increased, the density of GFAP-positive astrocytes increased markedly in the AH of the compressed half  MRI:  Cross sectional area (mm2)  Reduced in MC vs. control (p<0.05), SC vs. control (p<0.01), SC vs. MC (P<0.05) | KF:  Compression leads to increased GFAP+ astrocytes in the grey matter and anterior funiculi in the compressed half.  There was no difference in the lateral funiculi and dorsal funiculi between the compressed and contralateral halves.  Authors suggested that this provides evidence for the mechanistic basis of spinal cord damage that leads to transverse  lesion syndrome in unilateral compression myelopathy  L: No functional test  No cross validation of GFAP findings. |
| Zhao et al, 2005 (^75^) | Animals: Rabbits  Control= 3  Compression= 13  5- mild compression (MC) (2mm protrusion)  5- severe compression (SC)  (4mm protrusion)  Methods: Screw at C5 vertebral body  (anterior, unilateral)  Direction of compression: anterior  Duration of compression: 48 hours | Compression rate:  Less in mild CCC (24.3%) vs. severe CCC, (48.9%), p<0.01  Neurology:  MC: Normal neurology and gait  SC: All had tetraplegia and gait disturbance  Histology: H&E  MC: Proliferation of small inflammatory cells in AH, mild proliferation of reactive microglia in AH  SC: spinal cord deformed (reverse comma shape), cavity formation, bleeding, necrotic change in GM/ant funiculi, neuron loss around cavity  Immunohistochemistry:  RCA-1 (lectin *Ricinus communis agglutinin*-*1*)(microglia)  MC: mild reactive microglia proliferation.  SC: Marked reactive microglial proliferation ipsilateral to compression and moderate proliferation in contralateral half. | KF:  During severe unilateral compression there was marked proliferation of reactive microglia. Authors concluded that this provided evidence of resting microglia transforming into reactive microglia in early compressive myelopathy.  L: Duration of compression short  No information regarding how many were in MC or SC groups. |
| Penny et al, 2007 (^28^) | Animals: Texel and Beltex sheep  Control= 0  Compression=  10  Direction of compression: dorsolateral  Duration of compression:  Unknown | Sheep referred with signs of cervical spinal cord compression, confirmed with CT myelography followed by histology.  Myelography confirmed dorsal spinal cord compression in the region of C6-C7.  Histology: H&E  Myelin distension, presence of macrophages, and Wallerian degeneration (WD).  WD also in lateral funiculi, caudal to the site of compression. | KF:  Adipose polypoid projections in the dorsolateral region of C6-C7 caused chronic spinal cord compression with histology revealing evidence of Wallerian degeneration caudal to the site of compression.  L: Case series  Uncertainty regarding onset of DCM |
| Takenouchi et al, 2008 (^61^) | Animals: Twy/Twy mice  Controls= 10(5 ICR; 5 mice aged 1 month)  Compression= 10 mice aged 6m  Methods: Twy/Twy mice developed calcified deposits resulting in variable spinal cord compression at the level of C2-C3  Direction of compression: posterolaterally  Duration of compression: 6 months | Immunohistochemistry:  TUNEL staining  TUNEL  Increased TUNEL+cells in GM/WM at site of compression  Double Immunostaining (DIS):  Mitogen-activated protein kinase pathways –  1. (Apoptosis signal-regulating kinase 1 (ASK1)  2. Jun N-terminal kinase (JNK)  3. p38  MAPK pathways  1. Increased ASKI+ neurons and OLGs in GM/WM vs. control (p<0.01, p<0.01)  2. Increased JNK+cells in neurons and OLGs in GM/WM vs. control (p<0.01, p<0.01)  3. Increased p38+cells in neurons and OLGs in GM/WM vs. control (p<0.01, p<0.01)  Increased active caspase-3+ neurons and OLGs in GM/WM vs. control (p<0.01, p<0.01) | KF:  Mitogen-activated protein  kinase pathways including ASK1, JNK, and p38 were activated in destructive spinal cord under chronic compression  L: Study states increased presence of apoptotic signals transmitted from MAPK in OLGs and neurons, however MAPK inhibitors need to be used to determine significance of these signals. |
| Cheung et al, 2009 (^76^) | Animals= Sprague  Dawley Rats  Control= 0  Compression group= 5  Methods: Compression C5-C6 (lateral column) with urethane polymer  Direction of compression:  laterally  Duration of compression: 9 months. | Diffuse tensor imaging (DTI)  DTI confirmed polymer compressed the spinal cord laterally and the ipsilesional WM was distorted  Fractional anisotropy (FA)  - FA is a summary measure of microstructural integrity.  FA of ipsilesional region (IRA) lower than contralesional region (CRA)/ Dorsal region (DR) near epicenter (p<0.05))  Radial diffusivity (RD)  - RD increases in WM with demyelination. Changes in the axonal diameters or density may also influence RD.  RD of IRA higher than CRA near epicenter of lesion (p<0.05).  Axial diffusivity (AD)- In axonal injury AD decreases. Regions of interest (ROI)  AD: Lower AD near epicenter of lesion in IRA p <0.05  AD in rostral region increases farther away from epicenter  AD higher in rostral regions of DR, ventral region (VR) of CRA (p<0.05) and of IRA (p=0.102) | KF:  Feasibility of  using DTI to detect microstructural changes in chronic disease  has been demonstrated.  Near the ipsilateral epicenter of lesion, there is evidence of reduced microstructural integrity, axonal injury and demyelination.  L: No histology, immunohistochemistry or functional tests |
| Inukai et al, 2009 (^77^) | Animals: 45 Twy/Twy mice model  Controls= 11  Compression= 34  (Moderate compression [MC]=17;  Severe compression [SC]: 17)  Methods: Twy/Twy mice developed calcified deposits resulting in variable spinal cord compression at the level of C2-C3  Direction of compression: posterior  Duration of compression: 24-26 weeks range | Transverse remnant area of the spinal cord (TRAS): The ratio of the TRAS in the Twy/Twy mouse to that of the control designated as TRAS%  Moderate compression (TRAS 50-70%)  Severe compression (TRAS <50%)  Immunohistochemistry: TUNEL staining  Increased number of TUNEL+cells with increased severity of compression p<0.05  Double immunofluorescence staining (DIS): Most TUNEL+ cells were also RIP+ (marker of oligodendrocytes), this increased in the WM with increased severity of cord compression (78% in SC vs. 59% in MC).  Western blotting (WB)  TNF-[alpha]-Mediated Apoptosis  Overexpression of TNF-[alpha], TNFR1, and TNFR2, and activation of caspase-3, a marker of apoptosis seen in CCC vs. controls (no p values). Increase with magnitude of compression.  In sections double-stained for TNFR1, TNFR2, and activated-caspase-3 with RIP, a number of double-stained cells were identified among oligodendrocytes in the WM of SC  Transmission Electron Microscopy (TEM):  Maximum compression site showed apoptotic glial like cells in WM and accumulation of nuclear chromatin | KF:  There is increased number of apoptotic oligodendrocytes with increased severity of expression.  Overexpression of TNFa, TNFR1 and TNFR2 participate in apoptosis of oligodendrocytes.  L: No functional tests |
| Yu et al, 2009 (^32^) | Animals: Twy/Twy mice  Control= 4  Compression= 4  Methods: Twy/Twy mice developed calcified deposits resulting in variable spinal cord compression at the level of C2-C3  Direction of compression: posterolateral  Duration of compression: 4-5 months | Neurology:  Footprint analysis  - Forepaw and hind paw immersed in red and green dyes, respectively. Rats then walk across wooden board and footprint analysed.  Toe spread= Distance between 1^st^ and 5^th^ toe in forepaw (cm)  Reduced in CCC vs. control (p=0.001)  Interlimb coordination= distance between ipsilateral forepaw and ipsilateral hind paw  Increased in CCC vs. control (p=0.001)  Histology: H&E, LFB  CCC: AH/PH atrophy, demyelination  Immunohistochemistry:  NeuN+ (neurons)  CNP (oligodendrocytes)  GFAP (astrocytes)  Neurons  Reduced in CCC vs. control and +/- Twy mice control (p=0.001, p=0.005, respectively)  Increased NeuN+ neurons in uninjured rostral/caudal region (p=0.001, p=0.022, respectively)  Oligodendrocytes (OLG)  Reduced in CCC vs. control (p = 0.009). Increased in the uninjured rostral and caudal regions (p = 0.001 and p = 0.043, respectively) vs. control  CNP (found in OLG) expression reduced in CCC vs. control (p = 0.024).  Astrocyte  GFAP increased in CCC vs. control in epicenter (p = 0.001), caudal (p = 0.001) and rostral (p = 0.001).  TUNEL assay Increased number of TUNEL positive cell vs. control (p = 0.001).  FasL (Fas ligand) (transmembrane protein that belongs to tumour necrosis factor family. It binds to its receptor to induce apoptosis).  FasL expression  Increased FasL expression in ccc vs. control (p = 0.002).  Fas (cell death surface receptor)-mediated apoptosis  Fas expression  CCC: increased Fas expression in neurons (caudal) vs. ICR (p=0.003)  Increased expression in OLG vs. control (not significant)  Increased expression is astrocyte vs. control (p=0.001)  Increase in epicenter vs. ICR, (not significant)  Western blotting (WB):  microtubule associated protein (MAP2+) (neurons)  2',3'-Cyclic-nucleotide 3'-phosphodiesterase (CnPase) (oligodendrocyte)  Fas  Caspase 8  Neurons  Reduced microtubule associated protein (MAP2+) in C4 and C5 of CCC vs. control (P=0.006, P=0.002, respectively) and reduced NF200 vs. control in C4 and C5 of CCC (p=0.001, p=0.004, respectively)  Oligodendrocyte  CnPase reduced in CCC vs. ICR p=0.024  Fas protein expression was increased in CCC: 4months (p = 0.004) and 5months (p = 0.001) vs. control  Increased caspase 8 activation CCC vs. control (p = 0.002) and increased caspase 9 vs. control (p = 0.001) Increased caspase 3 (p = 0.049 and p = 0.049).  Immunoprecipitation (to determine interaction between Fas, FasL, and caspase-8)  Increased precipitation between Fas/FasL, FasL/pro-caspase 8, and caspase-8/Fas in ccc vs. control | KF: Chronic cervical spinal cord compression leads to Fas-  mediated apoptosis of neurons and oligodendrocytes, which is associated with activation of caspase-8, -9 and -3 and progressive neurological deficits.  L:  No cross validation of histological findings. |
| Hu et al, 2011 (^78^) | Animals: Sprague–Dawley Rats  Control= 0  Compression= 15  Rats categorized into three groups according to SEP responses  Group A= 6  Unchanged SEP    Group B= 5  Reduced amplitude (mV)  Group C= 4  Prolonged latency (ms)  Method: Polymer  Direction of compression: posterolateral  Duration of compression:  6m | SEP responses used to categorise groups  MR imaging  DTI- fractional anisotropy (FA)  Micro CT  Histology- H&E, LFB  Group A (Unchanged SEP)  - Compressed side – reduced FA (p<0.05) vs. uninjured side, less LFB staining, vacuolation in histology  Group B (Reduced amplitude)  Axon damage and demyelination in white matter seen at compressed side, indicated by lower FA (p<0.05) values under MR diffusion imaging and less LFB stain histologically (p<0.05) compared to other groups and uninjured side  Group C (Prolonged latency)  Loss of motoneurons in the grey matter vs. group A/B seen on histology (p<0.05) and injured side vs. non-injured side (p<0.05) and damage to the white matter indicated by lower FA (p<0.05) and less LFB staining. There was also higher contrast intensity in grey matter seen on the micro-CT.  All rats in all groups had central canal enlargement, intra-tissue bleeding and increased blood vessels in the grey matter, seen on histology | KF:  Categorising SEP responses by amplitude and latency may be useful in determining the extent  of ultrastructural damage of the spinal cord after chronic compressive injuries.  L: SEP and histological findings not correlated with neurological status. |
| Klironomos et al, 2011 (^7^) | Animals: 8 week old New Zealand rabbits  Control= 15  Compression group= 15  Methods: Compression achieved by implantation of aromatic polyether at the level of C7  Direction of compression: posterior  Duration of compression: 20 weeks | Neurology: Modified Tarlov Score (before op, immediately post op and 1 week post op)  0 No voluntary movements  1 perceptible movement at joints, hindlimbs follow  2 Good movement at joints but unable to stand up  3 Can stand up and walk, but unable to start running quickly  4 Normal  Neurology: Modified Tarlov Score  CCC: 3 (7/15), 2(8/15) vs. 4 in all controls  Histology:  H&E, Kluver-Barrerar (KB)  H&E  CCC: interstitial oedema, vacuolar degeneration  KB staining  Spinal cord oedema, myelin sheath fragmentation, axon swelling (myelin sheath enlargement), demyelination  Immunohistochemistry (IH)  Anti-neurofilament antibody (NF), anti-S-100 (astrocyte marker) antibody, anti-GFAP (astrocyte marker) antibody  NF: Lower expression in CCC vs. control p <0.001  - interstitial oedema, axonal swelling in CCC  S100: Higher expression in control vs. CCC (P=0.001)  - Significant demyelination in CCC  GFAP: Higher expression in CCC vs. control (stated significant but no p-value)  -Interstitial oedema in CCC | KF:  A new animal model of DCM was created, which shares similar characteristics to that of the human disease.  Chronic cord compression reduced levels of neurons but increased levels of astrocytes.  L: Location of demyelination not specified.  Location of neuron and astrocyte changes not specified.  No quantification of histological findings |
| Kubota et al, 2011 (^22^) | Animals: Male Sprague-Dawley rats  Control= 10  Compression= 10  Methods: Polyethylene line attached to a plastic plate was fastened to c4 vertebral body, line wrapped around spinal cord to cause compression  Direction of compression: anterior  Duration of compression: 12 months  All CCC rats developed myelopathy at 12 months | Neurology: BBB score at 3,6,9,12m  Control: 20 CCC: 17 P<0.05  Max treadmill speed (MTS) (seconds)  Control: 33 CCC: 21 P< 0.05  Sensory response frequency (%) using a Von Frey Filament  Forepaw (control =84%, CCC=26%) p<0.05  Hind paw (control= 83%hindpaw, CCC=21%) p<0.05  Histology: Nissl  CCC: Flatter/smaller/fewer neurons (GM), white myelin degeneration (WM)  MRI:  Cross sectional area: Control 14mm2 vs. CCC 9.3mm2  Compression ratio  Control 55% vs. CCC 31% | KF:  A new animal model of DCM was created, which shares similar characteristics to that of the human disease.  Chronic cord compression reduced neurological motor function and sensory function compared to controls.  L: Compression induced at C4  No quantification of histological findings. No cross validation of findings. |
| Kurokawa et al, 2011 (^44^) | Animals: Male Wistar rats aged 12- 14 weeks  Control= 14  Compression= 15  Methods: Compression between C5-C6 with polyurethane sheet  Direction of compression: posterior  Duration of compression: 12 weeks | Spinal cord blood flow (SCBF) (ml/min/100g))  using fluorescence microsphere technique  Increased in CCC (995) vs. controls (459), P=0.38 | KF:  Compression significantly reduces the blood flow in the compressed segment compared to controls.  L: No functional tests or histology. |
| Tanabe et al, 2011 (^47^) | Animals: Twy/Twy mice  Control= 10  Compression= 10  Methods: Twy/Twy mice developed calcified deposits resulting in variable spinal cord compression at the level of C2-C3  Direction of compression: posterolateral  Duration of compression: 24 weeks | p62 is a protein that interacts with the autophagic marker microtubule-associated protein 1 light chain 3 (LC3), and transports altered proteins to degradation by autophagy. p62 associates with LC3-II through its LC3-interacting region.  Histology: Nissl, LFB  Nissl stain: CCC: Reduced neurons in GM  LFB: CCC: axons are degenerated and demyelinated in WM  Immunohistochemistry (IH): performed to determine the cells containing and hypoxia inducible factor (Hif1a) and p62.  Hif1a- increased in nucleus  P62 detected in MAP2+ neurons, NF-H+ axons, GFAP+ astrocytes, olig2+OLGs  WB:  CCC:  Increased p62, ubiquinated proteins, LC3-1, LC3-II  Increased p62 in GM/WM at epicenter, caudal, rostral p<0.01  WB of free green fluorescence protein- (GFP) (indicator of autophagy flux)🡪 increased free GFP in neurons  EM: to visualize autophagosomes  CCC: Reduced axons, neurons exhibited autolysosomes (feature of autophagy), presence of autophagosomes in axons  Hypoxic stress induced using the CoCl2-chemical hypoxia model and anaerobic hypoxia model.  Hypoxic stress  In neuronal cells under hypoxic stress (not DCM) there is :  Increased Hif1a, GLuta1a  Increased polyubiquinated proteins  Increased p62  LC3 turnover assay (to examine levels of LC3-II expression in presence of lysosomal inhibitor, chloroquine).  LC3 turnover assay - chloroquine treatment increased the amount of LC3-II expression under hypoxic stress  Water-soluble tetrazolium salt (WST) assay (colorimetric assay to measure metabolic activity of viable cells. The absorbance obtained strongly correlates to the cell number).  WST assay  Reduced neuronal cells in normoxia and hypoxia (p<0.05; p<0.05) in CCC vs. control  3-methyladenine (3-MA), an autophagy inhibitor, decreased absorbance (number of neuronal cells) under hypoxic stress, p<0.01  Lithium chloride (LiCl), an autophagy activator, increased the number of neuronal cells under hypoxic stress (p<0.01) suggesting that autophagy promoted neuronal cell survival under hypoxic stress | KF: Autophagy promotes  neuronal cell survival under hypoxic stress suggesting that pharmacological inducers of autophagy may be  useful for treating degenerative cervical myelopathy patients  L: No histological quantification of findings  No functional tests |
| Lee et al, 2012 (^79^) | Animals: 75 female Sprague-Dawley rats  Control= 6  Mild compression (56.3%)= 27  Severe compression (87.4%)= 42  Methods: Titanium screw fixed extradural plate to cause compression at C6  Direction of compression: posterior  Duration of compression: 10 weeks | Neurology:  BBB  From 2- 10weeks, scores lower in SC than MC and control p<0.001  Control (21), MC (17), SC(11)  Inclined angle plane  (IAP°)  4 weeks post-surgery  MC (65) vs. control (80) p<0.001  SC (41) vs. control (80) p<0.001  MC vs. SC p<0.001  10 weeks post op- MC 78, SC 65  MC (78) vs. control (80), SC (65) vs. control p<0.001  MC vs. SC p<0.001  Grip strength (g)  Reduced grip strength between CCC and control (p<0.01) and between SC and MC (P<0.01)  C: 1220  MC: 659, 610, 743 (2,4,10 weeks post op)  SC: 393, 215, 517 (2,4,10 weeks post op)  Allodynia (Von Frey filament)- At 2 weeks post op  Mechanical allodynia Assessed by measuring the force required for the animals to withdraw the paw  Mechanical allodynia  At 2weeks post op, reduced withdrawal thresholds seen in all animals in MC/SC vs. control p<0.01. No difference between MC/SC.  Thermal allodynia  Assessed by measuring paw withdrawal latency in response to a focused beam of radiant heat  Thermal allodynia  No significant change in withdrawal latency between groups  Histology: Luxol fast blue (LFB), H&E  Total spinal cord areas (mm2) following compression: control (6.41), MC (4.43), SC (3) p<0.001  Total area of WM(mm2): control (4.45), MC (3.21) SC (1.35) p<0.001  Total area of GM(mm2): control (2.257), MC (1.152), SC (0.070) P<0.001  Immunohistochemistry (IH):  Neuronal nuclear antigen (NeuN) (Neuronal specific marker), ChAT (choline acetyltransferase), (marker of cholinergic neurons)  NeuN-+ at epicenter  Reduced in SC vs. control P<0.001  Control (1078.6), MC (416.0); SC (152.4)  ChAT + cells  Reduced in SC vs. control p<0.001  Control (40) MC (26) SC (5.3)  SEP  Peak amplitude (μV) less in MC(5.93)/SC(5.06) vs. control (42)  p<0.001  Increased peak latency (ms) in MC (2.68)/ SC(2.93) vs. control (1.69) p<0.001  Micro-CT  Compression ratio at 10 weeks:  Mild Compression (MC)- 56%  Severe compression (SC)-87% p<0.01 | KF: A titanium-screw-based chronic compression device (CCD) was designed to achieve progressive cord compression at the C6 level.  Severe compression led to worse neurological function and increased neuronal damage. |
| Uchida et al, 2012 (^80^) | Animal:  Twy/Twy mice  Control= 12  Compression= 28 (9 aged 16 weeks and 19 aged 24 weeks)  Methods: Twy/Twy mice developed calcified deposits resulting in variable spinal cord compression at the level of C2-C3  Direction of compression: posterolateral  Duration of compression: 16 and 24 weeks | Histology: LFB  CCC:  Spongy necrosis, myelin sheaths separation in anterior/lateral regions, axon swelling  Decrease in % cross sectional area of residual tissue vs. controls p<0.05  Immunohistochemistry:  TUNEL staining  Increased TUNEL+ cells in ant column aged 24weeks compared to 16 weeks in CCC p<0.05  Decreased TUNEL+cells in ant horn aged 24 weeks vs. 16 weeks (ns)  Double-staining: Tumor necrosis factor receptor 1 (TNFR1), cluster of differentiation 95 (CD95), and p75NTR (common neurotrophin receptor) with NeuN and RIP (oligodendrocyte marker) in the GM and WM of 24wks old mice  Reduced number of neurons (grey matter) in CCC vs. control. (No p value). However, expression of TNFR1, CD95, and p75NTR was mainly noted in OLG of white matter, no p value.  WB:  CCC: Increased apoptotic signals (TNFR1, CD95, and p75NTR) in CCC (24wks) vs. control p<0.05 | KF:  There is a significant correlation  between the proportion of apoptotic oligodendrocytes in the compressed area of the spinal cord and the magnitude  of cord compression.  Overexpression of TNFR1, CD95, and p75 NTR may play a role in the apoptotic process.  L: No quantification of histological findings |
| Hirai et al, 2013 (^49^) | Animals: Twy/Twy mice  Control= 29  Compression group= 29  Methods: Twy/Twy mice developed calcified deposits resulting in variable spinal cord compression at the level of C2-C3  Direction of compression: Posterior  Duration of compression: 12 weeks, 18 weeks, and 24 weeks | Histology: h&e  -The spinal canal and SC transverse area decreased with advancing age (p <0.01).  Immunohistochemistry (IH):  -M1 and M2 (microglial) phenotype markers increased with worsening age  -The CD11b-positive (microglia/macrophage marker) area increased with the worsening of CCC in GM & WM, especially AH, AC and LC (p<0.01)  -The NeuN-positive (neuron marker) area of GM lower in CCC vs. controls (P<0.01)  -% of microglia/macrophage of M2 phenotype = 82 at 18w, 61.6 at 24 week  -The no of resting microglia/macrophage lower in 24-week-old mice vs. controls p<0.01  -Increased duration of SCC🡪 increased neurotrophic factors and Mac-2(galactin-3)- a macrophage marker, which co-localised with M2 phenotype  -Increased CD4(T-helper cells) especially at 18 weeks vs. control p<0.01  Western blotting (WB):  Increased SCC, Increased Th1 cytokines (TNFa/IL-6) and BDNF/NGF/Mac-2 intensity  Increased TH2 cytokines (IL4, IL10, IL13) from week 18 | KF:  Spinal cord compression was associated with a temporal M2 microglia/macrophage response. There was a  persistent expression of Th1 cytokines and increased prevalence of activated M1 microglia/macrophages.  L: No functional tests |
| Karadimas, Klironomos et al, 2013 (^50^) | Animals:  New Zealand rabbits  Control= 15  Compression= 15  Methods: Aromatic polyether at C6  Direction of compression: posterior  Duration of compression: 20 weeks | Immunohistochemistry (IH):  Nuclear factor–[kappa] B (NF[kappa]B) - NF-κB subunits (p50 and p65)  These are transcriptional factors that contain the Rel family of proteins. The most common heterodimer in activated cells is the NF-κB1-RelA that contains the p65 and p50 proteins.  NF-κB binds to promoters of target genes involved in proliferation, apoptosis, vascular permeability, and neovascularisation.  Matrix metalloproteinase (MMPs) degrade extracellular matrix proteins, disrupt BBB, promote inflammation and demyelination  Urokinase-type plasminogen activator (u-PA) (MMP activator)  Increased cytoplasmic p65 (cp65) in CCC vs. control p<0.05  Increased nuclear p65 (np65) in CCC vs. controls p<0.05  Increased cp50 in CCC vs. p<0.05  Increased np50 in CCC vs. controls p<0.05  Increased MM9 in CCC vs. controls (p<0.05), increased MM2 in CCC vs. controls (p<0.05), increased uPA in CCC vs. controls (p<0.05), | KF:  There is a strong correlation  between the immunoexpression of NF- κB/p50, NF-  κB/p65, MMP-2, MMP-9, u-PA, and chronic cord compression.  L: No functional tests or histology. |
| Karadimas, Moon et al, 2013 (^22^) | Animals: Rats  Control= 4  Compression= 6  Methods: Aromatic polyether to promote calcification at C6  Direction of compression: posterior  Duration of compression: 6 weeks | Gait analysis (CatWalk)  Forelimb stride length FLSL (mm)  Hindlimb stride length HLSL (mm)  Base of support BOS (mm): distance between both hindpaws  Stance phase in forelimb (SPFL)  Stance phase in hindlimb (SPHL)  Reduced FLSL/HLSL in CCC vs. control p<0.001  Increased FL/HL stance in CCC vs. control p<0.01  BOS: Increased in CCC vs. control in week 4 (p<0.05) and continued to increase until week 10 (p<0.001)  SPFL and SPHL: Increased in CCC vs. control in week 4 (p<0.05) and continued to increase until week 10 (p<0.001)  Grip strength (g)  Reduced 4-10w post op in CCC vs. control p<0.001  Reduced withdrawal threshold in CCC vs. control: p<0.001  Tail flick test (TFT) for thermal hyperalgesia  Progressive reduction in withdrawal latency (s) in CCC vs. control. p<0.001  Histology: H&E, LFB  WM/GM degeneration, cystic cavitation, scar tissue was observed in CCC.  Immunohistochemistry (IH):  PKC-y (Corticospinal tract)  ChAT (motoneuron)  Monoclonal mouse anti-rat endothelial cell antibody 1 (RECA-1) and Evans blue (EB)- indicators of blood spinal cord barrier integrity  CCC:  Reduced PKC-y in dorsal column (p<0.05) vs. control 10 weeks post op  ChAT reduced in AH of GM vs. control (p<0.001)  RECA-1 - Reduced capillary density in GM and WM vs. control P<0.001 and increased Evans blue (EB) % vs. control P<0.001  TUNEL  CCC:  Increased TUNEL +cells vs. control p<0.001 10wks post op  Increased NeuN+TUNEL+cells vs. control p<0.001 10wks post op  Increased cleaved caspase-3 in CC1+ cells (p<0.001) vs. control 10wks post op  Increased NeuN/caspase 3+ cells p<0.001 vs. control 10wks post op  WB:  (Microglia/macrophage response)  Iba-1- increased in CCC vs. control 10w post op p=0.048  (Prop-apoptotic pathways)  Increased BCL-xl (p=0.048) in CCC vs. control 10wks post op  Increased Bax protein (p=0.044) in CCC vs. control 10wks post op  MRI  MRI Confirms posterior-anterior compression at C6  Compression ratio = 48 at 10 weeks post op  T1 intramedullary signal reflects cavity formation/glial scar formation  Electrophysiology  SEP  Hoffmann reflex +  (consists of two EMG responses, M wave (due to motor axon activation)and H wave (elicited by synaptic activator of motoneurons by muscle afferents).  SEP: Lower amplitude (mV) 10w post op in CCC vs. control p<0.005  Hoffmann reflex: Increased reflex (HMax/Mmax) 10 weeks post op in CCC vs. control p<0.05 | KF:  A novel rat DCM model was developed, which reproduces chronic and progressive nature of human DCM.  Chronic cord compression reduces gait stride length, grip strength and withdrawal latency vs. control. |
| Long et al, 2013 (^81^) | Animals:  36 adult Sprague-Dawley rats  Control= 6  Instant compression= 6 Group A: Gradual compression (maximum compression after 2 hours)= 12  Group B: Gradual compression (max compression after 24 hours)= 12  Methods:  Agarose Gel was  Turned into a water-absorbing  Polymer (1→4)-3,6-anhydro-a-l  galactopyranosyl-(1→3)—d galactopyranan  implanted into c5-c6  Direction of compression: posterolaterally  Duration of compression: 1 week | Neurology: BBB scores 1 week post op  Control: 21, A: 17, B:16  No significant decrease in CCC groups vs. controls or between group A/B  Histology: H&E, LFB  H&E stain: CCC (group A/B)  Reduced no of motor neurons in the anterior horn vs. control (p < 0.001), cytoplasmic reduction, [nuclear pyknosis](http://topics.sciencedirect.com/topics/page/Pyknosis), lower density of myelin sheath  No difference in speed of compression  LFB stain: CCC (group A/B)  PH and post funiculi, deformation, AH mild deformation. Reduced decussating fibres in grey matter. Vacuolar degeneration around axons. Reduced myelin sheath thickness.  No difference in speed of compression  MRI  NB Water and fluids appear dark on T1-weighted images (low signal) and bright on T2-weighted image (high signal).  Group A: Decreased signal in T1 and increase T2 signal suggesting intramedullary haemorrhage  Group B Compression occurred dorsally, no oedema/haemorrhage | KF:  The speed of compression is not a significant factor for establishing a reliable model for chronic cord compression if the compression is induced by gradual compression.  L: Maximum duration of compression was 1 week.  No sensory function or gait analysis  No immunohistochemistry |
| Takano et al, 2013 (^26^) | Animals: Twy/Twy mice  Control= 0  Compression= 5  Methods: Twy/Twy mice developed calcified deposits resulting in variable spinal cord compression at the level of C2-C3  Direction of compression: posterolateral  Duration of compression: 20 weeks | Diffuse Tensor imaging (DTI) for tract fibre (TF) analysis  The tract fibre (TF) ratio (the number of TFs at the C2–C3 level/the number of TFs at the C0–C1 level)  Severe cord compression occurred at C2-C3  Canal stenosis increased with age  Canal stenosis decreased TF ratio  Immunohistochemistry IH: RT-97 (normal neurofilaments)  SMI-31 (hyperphosphorylated normal axons)  Anti-RT-97 (neurofilaments) increased with TF ratio (p=0.0449)  Anti-SMI-31 (normal axons) increased with TF ratio (p=0.0489)  There was a positive correlation between the TF ratio and the RT-97–positive area (r = 0.7865, P = 0.0449, and SMI-31–positive area (r = 0.7746, P = 0.0489  Motor function:  Rotarod treadmill latency (RTL) and stride length (SL)  When TF ratio <0.09🡪 reduced RTL and SL  Compression>60% -🡪 reduced rotarod TML, SL  A lower number of RT-97- or SMI-31–positive fibres were associated with CCC.  The tract fibre (TF) ratio was strongly correlated with the RT-97/SMI-31–positive area and with motor function (rotarod latency, stride length). | KF:  DTI could be useful for detecting the early changes associated with CCC.  Reduced TF ratio was associated with reduced motor function.  CCC led to a reduction of normal neurofilaments and axons.  L: No functional tests or histology |
| Long et al, 2014 (^42^) | Animals:  Adult Sprague-Dawley rats  Control= 12  Compression group= 24  Methods: Compression at C6 with polyurethane sheet  Direction of compression: posterolaterally  Duration of compression: 4 Weeks | Neurology: BBB  Reduced score in CCC (18.74, 16.63, 15.22) vs. controls (20.87, 20.82, 20.89) at weeks 4, 12, 24 respectively (p<0.05)  Immunohistochemistry (IH)  -(CD34 microvessel staining used to calculate microvascular index (MVD)  Control: CD34-positive vascular endothelial cells sparsely distributed in GM  CCC: SC contour deformed, microvascular lumen narrowed and reduced no of microvessels in grey matter (GM) compared to control p<0.01  MVD:  Reduced MVD from ASA in CCC.  Micro-CT – used to measure Vascular Index (VI)  CCC: ASA disrupted, branches absent in the posterior and lateral funiculi  VI: Reduced at C6 in CCC vs. control P<0.01  There was significant correlation is also found between MVD and VI (r = 0.95, p < 0.01).  SEP  Latency (ms)  Control: 4.26 CCC: 8.47 (p<0.05)  Amplitude (uV)  Control: 7.17 CCC: 3.26 (p <0.05) | KF: Quantitative 3D micro-CT is a sensitive tool for investigating microvascular changes during chronic compressive spinal cord injury.  Chronic cord compression is associated with reduced neurological function. It is also associated with reduced number of microvessels and disruption of the anterior spinal artery.    L: No functional tests |
| Ma et al, 2014 (^82^) | Animals:  Rabbit  Control= 12  Compression= 36  Group A: compression until 3m  Group B: Compression until 6m  Group C: Compression until 9m  Method: Screw at C3  Direction of compression: anterior  Duration of compression: 3m, 6m, 9m | Neurology – Modified Tarlov. score  Control (5)  CCC: A (4) vs. control p<0.001  B(3.5) vs. control p<0.001  C(2.5) vs. control p<0.001  Group C worse than A p=0.013 and B p=0.017  Histology: H&E staining  Control: Normal  CCC: atrophy, neuron loss in AH, myelin/axon loss & vacuolation in lateral corticospinal tract. Changes worse with longer duration of compression  Demyelination worse in Group C vs. control  Immunohistochemistry:  MAP2+ neurons  CNP  TUNEL+ cells  Caspase 3  MAP2+Neurons  Reduced in CCC vs. Control p<0.001  No of neurons lowest in group C vs. group A/B (P <0.001, P=0.048, respectively)  Number of CNP positive cells in WM were smaller, with demyelination at the epicenter of compression, (no stats)  TUNEL+ cells  Increased no in GM/WM in group A/B/C vs. control (GM: p<0.001, p<0.001, p<0.001, respectively; WM p<0.020, p<0.001, p<0.001 respectively)  Increased in group C vs. A and B P<0.001, P<0.010, respectively)  Caspase 3+ cells  ↑ no in GM/WM in group A/B/C vs. control  (GM: P=0.026, P <0.001, P <0.001, respectively; WM: P=0.002, P <0.001, P <0.001, respectively).  ↑ in group C vs. A and B P<0.001, P<0.010 respectively  WB:  Optical density of caspase-3 bands ↑from group A/B/C vs. control (P=0.040, P=0.020, P <0.001, respectively). The highest band density appeared in the spinal cords from the 9-month.  Cortical SEP  Control: 16.85  A: 19.65 vs. control p<0.001  B: 20.90 vs. control p<0.001  C: 24.45 vs. control p<0.001  MRI T2-weighted SIR (signal intensity ratio)  (High SIR on T2-weighted MR images are associated with poor outcomes)  SIR in group A/B/C higher vs. control (P <0.001, P <0.001, P <0.001, respectively). 9-month had highest SIR vs. group A/B (P=0.010, P=0.020, respectively).  SIR in group A (1.08), B (1.15) and C (1.18) higher than control (1.00) (P <0.001, P <0.001, P <0.001, respectively).  SIR in group C had highest SIR scores vs. group A and B (P=0.010, P=0.020, respectively).  Spearman’s rank correlation test showed that there was close relation between MRI SIR and degree of caspase-3 expression in Western blotting (r=0.824. P <0.001) | KF:  T2 MRI could be used as a prognostic indicator of severity of apoptosis in DCM.  Chronic cord compression is associated with reduction in neurological function with evidence of neuron loss and myelin changes. There is also increase in apoptotic cells.  L: No functional tests |
| Takano et al 2014 (^83^) | Animals: Twy/Twy mice  Control= 0  Severe compression (SC)= 8 Mild compression (MC)= 8  Methods: Twy/Twy mice developed calcified deposits resulting in variable spinal cord compression at the level of C2-C3  Direction of compression: posterolateral  Duration of compression: 20 weeks  Canal stenosis ratio: 57% in SC  111.1% in MC | Neurology: Time on treadmill (s), TT  Forelimb stride length (FLSL)  Hindlimb stride length (HLSL)  TT: Increased in MC vs. SC p<0.05  FLSL: increased in MC P<0.05  HLSL: increased in MC P <0.05  Increased spinal cord area in MC p<0.001  Gene upregulation  Macrophage phenotype markers with RT-PCR  Increased TNFa (p<0.01), CD86 (P<0.05) in SC vs. MC  CD86/Iba+ cells increased in SC vs. MC P<0.01  No increase in arg1/CD163 (M2 phenotype)  Cyr61 increased in SC vs. MC (P<0.01)  Cyr61 located in compressed area and co-localised with reactive astrocytes  Histology: M1 inducer = cysteine rich protein 61 (cyr61)  Complement activated classic pathway  C1q leads to synapse loss and neuronal death  SC: Higher C1qa (p<0.01), c1qb (p<0.05), c1qc (p<0.01) vs. MC  C1q+ puncta were associated with synaptic protein (PSD-95).  Microarray analysis(MA)  Increased gene upregulation of following pathways:  Macrophage markers, Toll-like R signalling pathway, chemokine signalling pathway, prostaglandin synthesis and regulation, autophagy, CACP, apoptotic oxidative damage  Reduced gene upregulation in dopaminergic neurogenesis  In the severely compressed group; Iba-1 expression, TNFa, CD86 indicating M1 phenotype Cyr61, a chemotaxin for M1 macrophage recruitment, was expressed significantly more than in the mild compression group.  Microarray analysis of C1qa, C1qb, and C1qc revealed increased expression in the severely compressed group.  Arginase-1 and CD163 expression, indicator of M2 macrophage transition, was not significantly different.  CCC: Many C1q-positive puncta were co-localized with the postsynaptic protein PSD95  EM: CCC: There was extensive contact between presynaptic/postsynaptic elements and activated microglia/macrophage processes. | KF:  Severe cord compression leads to increase in complement mediated synapse loss and neuronal death.  L: No immunohistochemistry |
| Cheng et al, 2015 (^43^) | Animals: Rats  Control= 24  Compression= 24  Methods: Compression at C6 with polyurethane polymer  Direction of compression: posterolateral  Duration of compression: Analysis from day 1 to day 70 | Neurology: BBB score  -Decreased score 28^th^ day post op vs. control (p <0.05)  -Increased score at day 70 compared to day 28 (p <0.05)  Micro-CT to investigate spatial and temporal changes of ASA and ARA in compressed spinal cord  Vascular morphology with micro-CT  CCC: Decrease of anterior spinal artery (ASA) diameter from day 28 to 70 vs. control (p<0.05) with a maximum decrease at day 42 followed by a marginal increase up to day 70  CCC: Decrease of anterior radicular artery (ARA) diameter vs. control (p<0.05); maximum decrease at day 28 followed by an increase at day 42 and decrease at day 70.  No change in VA(vertebral artery) diameter vs. control  Vascular Index (measure of vascular network)  -CCC: Decrease in VI at day 28 vs. control (p<0.05)  -CCC: Increase in VI at day 70 vs. day 28 (p<0.05), similar level to control  SEP  -Reduction in amplitude and prolongation of latency in compression group vs. control at day 28 (p<0.05)  -Improvement in SEP at day 70 compared to day 28 (p<0.05) | KF:  Chronic cord compression leads to a decrease in diameter of the anterior spinal artery and anterior radicular artery.  L: No histology/ immunohistochemistry |
| Jiang et al, 2016 (^11^) | Animals:  Male adult goats  Controls= 6  Compression=  A: Acute cord compression: 6  B: Anterior CCC (1 death during anaesthesia): 6  C: Anterior CCC + ACC: 6  Methods: Progressive screw compression at C5  Direction of compression: Anterior  Duration of compression: 5 weeks | Neurology: Modified Tarlov Score (post op 5d, 2w, 5w, 8w)  0 Hind leg paralysis  1 Able to move hindlegs but not stand  2 Stand on hindlegs  3 Can walk but not run  4 Can run but not jump  5 Normal gait and can jump  CCC: Lower score (4.4, 4.2, 3.7, 3.5) vs. control (5,5,5,5) post op 5d, 2w, 5w and 8w (ns)  Histology: CCC (group B)  Partial WM demyelination, neuronal degeneration, and necrosis.  Reduction of nerve cells  EM: CCC (group B)  -Axon swelling, thinning medulla sheath with loosening surfaces, and uneven complete disintegration.  -Some mitochondria had less cristae and vacuolar degeneration. -Severe swelling in the microvascular endothelial cells.  -Increased nuclear heterochromatin and vacuoles in intracytoplasm.  SEP  Amplitude  Reduced in CCC (0.84) vs. control (3.15) 5 weeks post op p<0.05  Latency  Prolonged in CCC (19.76) vs. control (12.76) 5 weeks post op p<0.05 | KF:  Chronic cord compression leads to progressive reduction in neurological function with increased duration of compression.  There is also evidence of axonal disintegration and neuronal degeneration.  L: No immunohistochemistry |
| (Satkunend-rarajah et al, ^84^) | Animals:  ntSCI mice,  Control: 7  ntSCI: 7  Direction of compression: un specified  Duration of compression: 4, 8 weeks  Animals:  VGLUT2- PSAM mice  control: 7  VGLUT2- PSAM: 6  Direction of compression: unspecified  Duration of compression: 4 weeks, 8 weeks  Animals:  VGLUT2- PSAM mice  Control: 10 VGLUT2- PSAM mice before SCI  VGLUT2- PSAM mice after SCI: 10  Direction of compression: unspecified  Duration of compression: 4 weeks  Animals:  VGLUT2- hM3Dq mice  Control: 8  VGLUT2- hM3Dq mice: 9  Direction of compression: unspecified  Duration of compression: unspecified | Changes in ntSCI mice compared to controls  Plethysmography:  28.6% reduction in inspiration duration (P = 0.0015, 95% CI = -0.038 to -0.012)  Immunohistochemistry:  64% loss of PMN (CTb)- traced  Increase in excitatory VGLUT2+bouton in surviving PMN population by 5.4 times compared to control (P= 5.56 x 10^-8^, 9% CI= -41.71 to -28.29)  2.75 fold increase in interneurone connection to PMNs in ntSCI mice compared to controls (P=2.0 x10^-4^, 95% CI = 96.44 to 183.8, unpaired t-test, two tailed)  Selective silencing of mid cervical eINs in ntSCI mice:  Plethysmography:  Increase in average number of hypopnea events from 5.24% to 61.17% (P = 2.78 x 10^-5^, repeated- measures ANOVA) after 8 weeks.  EMG:  Mid-cervical eIN, in ntSCI mice, can maintain diaphragmatic function in absence of ipsilateral bulbospinal input, after C2 hemisection. Ipsilateral diaphragmatic activity and PMN activity was terminated if eIN activity was blocked.  Administration of PSEM ligand of PSAM, to block eIN activity in VGLUT2- PSAM mice: PSEM administered to post SCI VGLUT2- PSAM mice, 4 weeks after traumatic SCI, caused 69.5% lower mean amplitude compared to baseline.  Inducing cervical eIN activity immediately after C2 hemisection:  EMG:  Immediate motor recovery of ipsilateral hemi diaphragm to 41.78% and to 47.5% of contralateral EMG amplitude and area. (P = 8.23 x 10^-5^ for amplitude and area, two sided Mann- Whiteney- U test) | KF: Excitatory interneurones are recruited through plasticity in ntSCI mice to maintain ventilatory function by forming connections with remaining PMN. eIN not necessary for breathing in normal mice.  Limitation:  Low n number for the different studies.  Does not specify direction of compression in ntSCI, VGLUT2- PSAM mice models |
