# Supplementary material for: The Pathophysiology of Degenerative Cervical Myelopathy and the Physiology of Recovery Following Decompression
Source: Front Neurosci. 2020 Apr 30;14:138. doi: 10.3389/fnins.2020.00138 (PMC7203415; doi:10.3389/fnins.2020.00138)
Supplement: Supplementary file 2 [file Table_2.docx]

**Table 2: Pathophysiology of DCM (studies in compressed cords with therapeutic intervention)**

| **Authors** | **Methods** | **Results** | **Key Findings (KF)**  **Limitations (L)** |
| --- | --- | --- | --- |
| Xu et al, 2006 (^56^) | Animals: Twy/Twy mice  ICR Controls (+LacZ)= 15  Compression group total= 27  Compression + BDNF= 12  Compression +LacZ= 15  Methods= Twy/Twy mice developed calcified deposits resulting in variable spinal cord compression at the level of C2-C3  Direction of compression: Posteriorly  Duration of compression: 16 weeks | LacZ marker gene using adenoviral vector (AdV-LacZ) was used to evaluate retrograde delivery from the sternomastoid muscle in adult Twy/Twy mice (16-week-old)  and (control).  Histology: Nissl (to detect anterior horn neurons)  Nissl staining reduced in CCC vs. control (no p values), increased with CCC+BDNF (p<0.05)  Immunohistochemistry: anti-Brain derived neurotrophic factor (BDNF), anti-ChAT, and anti-AChE antibodies  Retrograde gene delivery treatment (AdV-BDNF)  ChAT neurons reduced in CCC vs. control (no p values), increased with CCC+BDNF (p<0.05)  AChE neurons reduced in CCC vs. control (no p values), increased with CCC+BDNF (p<0.05) | KF:  Targeted retrograde AdV-BDNF-gene  in vivo  delivery may enhance neuronal survival un-  der chronic mechanical compression.  L: Statistical analysis incomplete |
| Uchida et al, 2008 ^(60)^ | Animals: Twy/Twy mice  Controls= no specific numbers provided Gene delivery group= 93 (16w, n= 75, 20w, n=18)  Methods= Twy/Twy mice developed calcified deposits resulting in variable spinal cord compression at the level of C2-C3  Direction of compression: Posterolaterally  Duration of compression: 16 weeks | Histology: Nissl (Cresyl violet)  Immunohistochemistry: anti-NT-3, anti-ChAT, and anti-(Neurotrophic tyrosine kinase receptor ) trkC antibodies  Retrograde gene delivery treatment (AdV-NT-3)  Transfection of replication-defective AdV vector encoding [beta]-galactosidase (AdV-LacZ) used to demonstrate delivery to target region and therefore can be used as a marker for compressed group without treatment.  Histology: H&E showed compression more compression in 20 w mice vs. 18 w mice.  IH: Control (Adv-LacZ)- lower Nissl, ChAT and trKC in 20w vs. 16w.  Reduced motoneurons as evidenced by reduced Nissl staining, ChAT and TrKC levels in compressed group (no p values). Increased Nissl, ChAT, and trkC positive neurons with AdV-NT3 positive vs. compression (P<0.05)  CCC: Reduced neuron soma, length of neuritis and branch length- increased significantly by AdV-NT3 (p<0.05, p<0.05, p<0.05) | KF:  Targeted retrograde NT3 -gene  in vivo  delivery may enhance neuronal survival un-  der chronic mechanical compression.  L: No cross validation of findings  Numbers of control unclear |
| Kurokawa et al, 2011 (^44^) | Animals:  Male Wistar rats  Group A: Control + saline= 6  Group B: Control+ 200ug/kg Limaprost = 6 (prostaglandin PGE1 derivative- vasodilator, antiplatelet agent)  Group C: CCC = n=15  Group D: CCC+ Limaprost= 15  Methods: Polyurethane sheet C5-C6  Direction of compression: posterior  Duration of compression: 26 weeks | Forced locomotion capability (FLC) on a rotating treadmill  Reduced FLC in group C at week 6 compared to control p<0.0001  Histology: H&E  Cord contour flattened in group C  Reduced number of neurons in group C vs. controls (p=0.027) and increased in group D (p=0.027) | KF:  Chronic cord compression led to a decrease in locomotion and neuronal loss. The use of a prostaglandin vasodilator attenuated the loss of neurons.  L: Full functional test not performed  No cross validation of findings |
| Yu et al, 2011 (^15^) | Animals: Twy/Twy mice  Control= 12  Compression= 12  Methods: Twy/Twy mice developed calcified deposits resulting in variable spinal cord compression at the level of C2-C3  Direction of compression: posterolaterally  Duration of compression: 4 weeks | Neurology:  Toe spread  CCC leads to reduced toe spread vs. control, Anti- FasL Improves % toe spread in IgG treated mice (p<0.003) and saline control (p=0.001) at 3 weeks.  Immunohistochemistry:  Iba1+ (microglia/macrophage), GFAP, NF200 (neurons), BIII tubulin (axons)  Fas-mediated apoptosis assessed with delivery of Anti-Fas-ligand to reduce Fas expression in Iba1+, caspase 9, neurons, Bcl-2 (anti-apoptotic protein)  :  Increased Iba1+, GFAP  Reduced NF200, BIII tubulin  Anti- FasL:  -reduces Iba in CCC vs. saline control (p=.0.003) and IgG control p=0.004,  -reduces GFAP in CCC vs. saline control (p=0.0048)  -reduces caspase9 in CCC vs. control p=0.026  -reduces loss of neurons in CCC vs. IgG control (p=0.019) and reduces loss of axons vs. IgG control (p=0.002) and saline control p=0.037  -Increases Bcl-2 in CCC vs. IgG control (p=0.001), saline control (p=0.007), ICR control (p=0.002) | KF:  Chronic cord compression reduces locomotor function, which was reversed by the use of Anti-FasL.  Anti-FasL also attenuated the loss of neurons and axons seen in chronic cord compression.  Targeting the Fas death receptor pathway may thus be a viable neuroprotective strategy to attenuate neural degeneration and optimize neurological recovery in DCM.  L: No cross validation of findings |
| Wang et al, 2012 (^25^) | Animals:  Rats  Controls= 5  Compression=  Mild compression (n=5) with smaller polymer 1.5x0.7x0.3mm3  Severe compression (n=5) with polymer size 5x1.5x0.7mm3  Methods: Polymer (made of hydrogel, composed of polyvinyl alcohol and polyacrylamide)  Direction of compression: posterior  Duration of compression: 4 weeks | Neurology: Arbit’s grading  Grade:  0= normal  1= hip instability  2= mild weakness- able to run  3= moderate weakness—able to walk but not run  4= marked weakness—can stand but not walk  5= severe weakness—animals cannot stand and movement in the hind limbs is slight;  6= paraplegia  Inclined plane test (ability to maintain postural stability)- placed on an inclined plane, and the maximum inclination at which the rat could maintain its position for 5 s was recorded as the final angle.  Neurology  During the first 4 weeks, MC and SC had higher grades vs. control (p<0.05) followed by gradual reduction.  Before 24 weeks, the two compressed groups showed a significant higher grade than did the sham group (P<0.05).  IAP  Max IAP of control =80degrees  IAP lower in MC and SC vs. control at 24 w (p<0.05)  Histology: LFB  Reduced spared white matter in MC vs. control (p<0.05), SC vs. control (p<0.05) and SC vs. mc (p<0.05)  Immunohistochemistry:  NeuN (Neurons)  RIP (OLG)  MBP (axons)  NFkB (transcription factor)  C-IAP2 (apoptosis inhibitor)  BDNF^+^ and vascular endothelial growth factor (VEGF)  -Reduced neurons in epicenter of compression - MC vs. control (p<0.05), SC vs. control (p<0.01) and SC vs. MC (P<0.05)  -Reduced OLG in MC vs. control (ns), SC vs. control p<0.01, SC vs. MC p<0.05,  -Higher MBP+ cells and higher density of axons at 28w in SC vs. 4w in SC  -Decreased periaxonal space (gap between axolemma and myelin sheath- sign of axon pathology) at 28w SC vs. 4wk SC.  -NFkB and C-IAP2 co-expressed in neurons and OLGs  -Increased in compressed groups (which group not specified) vs. control p<0.05  BDNF^+^ and VEGF^+^ cells in the astrocytic culture was significantly higher in the MC and SC groups than that in the control after 4 weeks post-compression (P<0.05)  After 1000 μg/ml of hyaluronan tetrasaccharide (HA_4_) VEGF and BDNF were significantly upregulated in vitro (p<0.05)  Western blotting:  NF200 (neurons)  MBP (OLG)  NFkB  C-IAP2  Caspase-3  WB:  -Reduced NF200 in MC vs. control (p<0.01), -SC vs. control (p<0.01), SC vs. MC (p<0.05) -Reduced levels of MBP in MC vs. control (p<0.05), SC vs. control (p<0.01), SC vs. MC (p<0.05)  -Increased NFkB in MC at 4w vs. control p<0.05, increased NFkB in SC at 4w vs. control p<0.05  -Increased C-IAP2 at SC vs. MC p<0.05  -Caspase-3 expression in the 28 widely compressed group was significantly lower than the 4 week widely compressed group P<0.05  Effect of HA_4_ on neuron cell viability assay  (To investigate the effect of Hyaluronan tetrasaccharide (HA_4)_ on the inhibition of H_2_O_2_-induced apoptosis, using an MTT assay)  HA4 at doses of 100ug/ml and 1000ug/ml increased levels of NFkB and C-IAP2 vs. control  Cell viability assay  -HA4 at doses of 100ug/ml and 1000ug/ml reduced H202 induced apoptosis (p<0.05, p<0.05)  ELISA and MALDI-TOF mass spectroscopy  Enzyme-linked immunosorbent assay (ELISA) and MALDI-TOF (Matrix Assisted Laser Desorption/Ionization- ime-of-flight mass spectrometer) mass spectrometry to analyse levels of hyaluronan in CSF  Increased levels of HA in CSF (uG/ML) in SC vs. MC, SC vs. control, MC vs. control (p<0.05, p<0.05, p<0.05, respectively)  Electrophysiology  Motor evoked potentials (MEPs) - recorded from tibialis anterior muscle and the reference electrode inserted into the footpad.  For sensory function assessment, SEP were elicited by applying stimuli to the tibial nerve at the ankle  MEP  Prolonged latency in MC at 4weeks vs. MC AT 28 W (P<0.05) R+L  Prolonged latency in SC at 4weeks vs. SC AT 28 W (P<0.05) in R  Increased amplitude in MC at 28w vs. MC at 4w (P<0.05)  Increased amplitude in SC at 28w vs. SC at 4w (P<0.05)  SEP  Prolonged latency in MC at 4weeks vs. MC AT 28 W (P<0.05)  Prolonged latency in SC at 4weeks vs. SC AT 28 W (P<0.05) in both R/L  Increased amplitude in MC at 28w vs. MC at 4w (P<0.05)  Increased amplitude in SC at 28w vs. SC at 4w (P<0.05) | KF:  Chronic cord compression causes increased severity of weakness.  Hyaluranon - HA4 in the CSF may increase levels of NF-KB, c-IAP (apoptosis inhibitors) and neurotrophic factors in chronic cord compression to enhance behavioural recovery.  L: Study states there are 5 rats per group (Control, mild compression and wide compression, however this does not equate to 60)- were some results not analysed? |
| Moon et al, 2014 (^33^) | Animals:  female Sprague-Dawley rats  Control= 6  A Compression + saline= 18  B Compression + Riluzole= 17  Methods: Compression with screw through a rod fixed to spinous processes of C2 and T2  Direction of compression: posterior  Duration of compression: 8 weeks. | Neurology: Mechanical allodynia (MA)  Von Frey filaments applied to mid-plantar surface of forepaws and hindpaws.  5 filaments were applied in ascending order, and the smallest filament that elicited a positive response was considered the threshold stimulus (reduced threshold=increased sensitivity)  MA- forepaw  Increased sensitivity in CCC + saline vs. control at weeks 3- 8 p<0.001  Increased sensitivity in CCC + Riluzole vs. control at weeks, 4, 5 (p=0.002; p=0.018). Reduced sensitivity in CCC + Riluzole vs. CCC + saline at week 8 p<0.001  MA-hind paw  Increased sensitivity in CCC+ saline vs. control at 2-8 weeks post (p<0.001)  Increased sensitivity CCC + Riluzole vs. CCC + saline at 4 weeks post surgery (p=0.001)  Tail withdraw latency (TWL) to thermal noxious stimuli  Dorsal surface of the tail exposed to a beam of light generated from an automated analgesia meter. The timer was stopped when the animal flicked its tail away from the beam of light. Tail-flick latency was obtained over 3 trials. The mean latency was used as a measure to indicate thermal hyperalgesia.  Reduced TWL in CCC vs. control (o<0.05) at 8weeks.  CCC + Riluzole increased TWL at 8 weeks vs. CCC + saline (p<0.05).  Gait (CatWalk system) 8 weeks post op- Horizontal glass plate and video capturing equipment underneath connected to a computer. Measurement of swing phase duration, swing speed and paw intensity (mean pressure exerted by paw during floor contact)  CCC + Riluzole increases swing phase of forelimbs (<0.05) and hindlimbs (p<0.05)  CCC + saline – reduces paw intensity vs. control (p<0.05) – Riluzole increased paw intensity P<0.001)  Histology: H&E/LFB  CCC🡪 Increased % scar tissue vs. controls, (reduced by Riluzole p=0.033)  CCC + Riluzole- Increases grey matter vs. CCC + saline (p=0.002)  Immunohistochemistry:  Iba-1 immunoreactivity  CCC 🡪 increases Iba-1, increases soma and short processes indicating activated microglia state p<0.001; reduced by Riluzole (p=0.001)  CCC🡪 increases galactin-3 (present in activated microglia) expression vs. controls p<0.001; reduced by Riluzole p=0.013  Increased GFAP in CCC + saline vs. control=p<0.001; Riluzole reduces this increase p=0.027  The NMDA receptor subunits (pNR1 and pNR2B) has been implicated in development of neuropathic pain (Gao et al, 2005) thus authors tested this in the DCM model.  CCC🡪 increased pNR1 (p=0.006) and pNR2B+ cells (p<0.001) in dorsal horns, both reduced by Riluzole (p=0.006 vs. p<0.001 respectively)  pNRI and pNR2B co-localised mainly in astrocytes and some neurons of dorsal horns.  Micro-CT (%)  (1-2c/(a+b)) x100  C= AP canal diameter at level of max compression  A= AP canal diameter at nearest normal level above site of compression  B= AP canal diameter at nearest normal level below site of compression  Micro-CT- Compression ratio  CCC: 30%  CCC + Riluzole: 32%  Control (sham)= not measured  No significant difference p=0.78 | KF:  Chronic cord compression leads to persistent mechanical allodynia and thermal hyperalgesia.  Authors suggest Riluzole reduces neuropathic pain in DCM via reduced GFAP immunoreactivity and reduced pNR1 and pNR2B levels.  L: Compression induced between C2-T2 |
| Yamamoto et al, 2014 (^40^) | Animals:  Male Wistar rats aged between 12 and 14 weeks  A: Control= 7  B: Control + cilostazol (Type 3 PDE inhibitor)= 7  C: Compression + vehicle solution= 13  D: Compression + cilostazol= 13  Methods: Urethane compound polymer inserted into C5-6 epidural space.  The sheet expands for 48-72 hours at which it reaches constant volume.  Direction of compression: posterior  Duration of compression: 25 weeks | Neurology: Behavioural test: Foregrip strength (FGS) (right and left)  Rats given 1 second to establish a grip on the handled of a digital force metre and then pulled backed until grip released. FGS strength measured when rat gasped bar back immediately with all fingers and upon release paws were relaxed and not clenched.  FGS  Reduced at 7 weeks (R) and 8 weeks (L) vs. control p<0.05. Bilateral transient reduction in FGS after surgery in group D, but preserved thereafter.  Forced locomotion capability (FLC) (f  Forced running capability on a rotating treadmill)  FLC:  Significant reduction at 19weeks and thereafter in group C (P<0.05). No decrease in FLC group D.  Histology: H&E  Histology:  Group C&D:  Sinusoidal dilation of veins  GM: cavitation  WM: Cavities, myelin debris in anterior/lateral/posterior columns  Motoneuron numbers:  Reduced in group C vs. control (p<0.05) Neuronal loss prevented in group D (p<0.05)  Immunohistochemistry:  TUNEL+ cell  No TUNEL+ cells seen in controls.  TUNEL+ cells present especially in WM in group C  The number of TUNEL-+ cells in group D lower in GM/WM (stated significant, no p values) | KF:  Chronic cord compression reduces neurological function, increases neuron loss and apoptosis. Cilostazol, a selective Type III phosphodiesterase inhibitor preserves neurological function and neurons.  L: No cross validation of neuron numbers |
| Yoshizumi et al, 2016 (^20^) | Animals:  rats  Control= 12 (group A)  Compression+ NS=12 (group B)  Compression= G-CSF= 12 (group C)  Methods: Urethane polymer at C5-C6  Direction of compression: posterior  Duration of compression: 26 weeks | Prevention experiment  Granulocyte colony-stimulating factor (G-CSF)/NS administration started immediately post op  Rotarod  Rotarod- Reduced duration of walking in group B vs. A/B p<0.001  Group C similar to A  Grip strength (GS)  Grip Strength: Reduced in group B vs. A/V p<0.001  Histology:  Histology: Reduced neurons in AH 26w post op p<0.001  Immunohistochemistry: TUNEL  IH: TUNEL- TUNEL:DAPI+ cells higher in B vs. A (p<0.05) and C (P=0.057)  Treatment experiment  G-CSF/NS administration started 8 weeks post op  Treatment experiment  Rotarod  Reduced duration in B vs. A 6w post op p<0.05  Group C increased vs. B from 9 -15 w p<0.05  Grip strength:  Reduced in group B vs. group a from 8w P<0.05  Improved in C vs. B from 9 weeks post op p<0.05  Histology:  Motor neurons in AH: reduced in B/C vs. A p<0.001  Increased in C vs. B p=0.142 | KF:  G-CSF prevents the decline in motor functions and preserves motor  neurons in the rat chronic cord compression model. G-CSF also improves motor  function in the progressive phase of compression myelopathy  L: No cross validation of neuron numbers seen in histology |
