# Supplementary material for: The Pathophysiology of Degenerative Cervical Myelopathy and the Physiology of Recovery Following Decompression
Source: Front Neurosci. 2020 Apr 30;14:138. doi: 10.3389/fnins.2020.00138 (PMC7203415; doi:10.3389/fnins.2020.00138)
Supplement: Supplementary file 3 [file Table_3.docx]

**Table 3: Studies investigating physiological and pathological changes following decompression**

| **Authors** | **Methods** | **Results** | **Key Findings (KF)**  **Limitations (L)** |
| --- | --- | --- | --- |
| Harkey  et al. 1995 (^29^) | Animals:  Mongrel dogs  Compression= 6  Decompression= 6  Methods: Compression with Teflon screw  Direction of compression: Posterior Teflon washer and anterior Teflon screw  Duration of compression: 45 weeks  Decompression: Removal of screw  Animals sacrificed between 44-77 weeks | Neurology:  Decompression group (dCCC): 6/6 improved between 2-5 weeks post op  2/6 had unilateral deficits  SEP  dCCC: 2/6 recovered latency  SCBF: Hydrogen clearance method  CCC: Reduced blood flow immediately following compression  dCCC: No significant changes in blood flow between two groups  MRI:  dCCC: 5/6 had high intensity signals pre op and 1/6 had low intensity signals preoperatively. Repeat MR revealed decrease in signal and size of lesions in 3/6 animals post op.  CCC: 1^ST^ MR- 5/6 had high intensity abnormalities, 2^nd^ MR- reduced signals/size of lesions in 2/6 animals.  Histology:  H&E, LFB  Well stain for myelin  Fink and Heimer method (degenerated axons in wm/gm)  GFAP+ fluorescence (glial responses)  CCC:  1/6 frank cavitation  1/6 mild large motor neuron cell loss  3/6 motor neuron cells loss, oedema, necrosis  dCCC:  3/6 motor neuron cell loss, necrosis, and cavitation.  1/6 mild, large motor neuron cell loss  2/6 no pathological abnormalities  One animal had mild, large motor neuron cell loss only, and two had no pathological abnormalities  No WM demyelination/axonal degeneration  In animals with cavitation of GM. slight degrees of demyelination appeared in the ventral funiculi or in the dorsal part of the lateral funiculi.  No difference in the degree of demyelination or axonal degeneration seen between groups. | KF: All animals in the decompressed group showed significant neurological improvement after decompression.  L: Level of cervical spinal cord compression was not specified.  No immunohistochemistry |
| Karadimas et al, 2015 (^56^) | Animals:  Rats  Compressed group  Decompressed group  Methods: Aromatic polyether polymer implantation at C6  Duration of compression: 6 weeks  Direction of compression:  Therapeutic:  Riluzole 8mg/kg administered at 4 weeks after polymer implantation and continued for 2 weeks post decompression | Neurology:  CatWalk  Von Frey Filament  Tail Flick  Hand grip strength  Decompression leads to reduced forelimb stride length (FLSL), vs. compression p<0.05, Riluzole improves FLSL vs. decompression alone p<0.05  Riluzole improves regulatory index (%) after decompression vs. decompression alone p<0.05  Riluzole doesn't change any other parameters  dCCC+R reduces neuropathic pain vs. control p<0.05  SCBF: Using MRI Flow sensitive attenuating inversion recovery (FLAIR)  CCC: 74.33/100g/min. dCCC: 167.83/100G/min p<0.05  Immunohistochemistry: NeuN, ChAT, PKC-y, GFAP, Iba-1, JC-1 (mitochondrial membrane potential), 8-oxoG (Increased in reactive oxygen species)  ROS- Cobalt chloride, stabilises HIF-1a in normoxia- increases ROS- resembling ischemia-perfusion injury  IH:  DIS: NeuN: 8-oxoG  Decompression alone leads to neuronal survival with expression of 8-oxoG DNA vs. control p<0.05  Riluzole reduces this vs. decompression alone p<0.05  CCC reduces ChAT neurons vs. control p<0.05, dCCC increases levels vs. CCC p<0.05, dCCC+R increases further vs. dCCC p<0.05  Riluzole promotes axonal preservation area of most CST vs. control p<0.05, dCCC promotes axonal preservation vs. CCC p<0.01  dCCC+R promotes axonal regeneration further, more than Riluzole alone p<0.01 or dCCC alone p<0.01  CCC- increases Iba-+ cells in lumbar dorsal horns, dCCC reduces Iba+ cells vs. CCC p<0.05, Riluzole reduces Iba+ cells vs. CCC p<0.05. dCCC+R leads to greater reduction in Iba+cells p<0.05  ROS  Riluzole reduces 8-oxoG DNA positive cells (43.9%) vs. decompression alone (65%) p <0.05  Riluzole reduces depolarisation of mitochondrial membrane potential from 35% (in CoCL) to 5% p<0.0.5 | KF: Spinal cord blood flow  measurements increased after decompression  surgery in rats. DCM rats showed a transient postoperative  neurological decline akin to that seen in some DCM patients, suggesting that ischemia-reperfusion injury may occur after decompression surgery. Riluzole treatment attenuated oxidative DNA damage in the spinal cord and postoperative decline after decompression surge  L: No consistent numbers across different studies |
| Dhillon et al, 2016 (^18^) | Animals:  Rats  Control= 5  Compression group= 5  Decompression group= 5  Method of compression: Polyurethane elastomer at C/C4  Direction of compression: Posterior  Duration of compression: 10 weeks  Duration of decompression: 5 weeks | Neurology  BBB  Reduced scores in CCC vs. control p<0.0001m improved by decompression after 3 weeks p<0.0001  Forepaw and hind paw slips  Forepaw stride length and width  Forepaw/hind paw slips  CCC led to increase in FP and HP slips vs. control (?p value), decompression decreased FP slip (p<0.001) and HP slips (p<0.0001)  IH: synaptophysin, GAP-43, GFAP, Iba-1, Olig-2, APC, caspase-3  IH:  Increased caspase 3 in CCC vs. control at epicenter/caudal and rostral sites p<0.0001  Decreased caspase 3 in dCCC approaching normal levels p<0.0001  Increased APP+ axons in white matter in CCC vs. control at epicenter/caudal and rostral sites p<0.0001  Decreased APP in dCCC approaching normal levels p<0.001  Increased APP+ plaques in grey matter in CCC vs. control at epicenter/caudal and rostral sites p<0.05  dCCC did not decrease levels of APP+ plaques in GM.  Reduced 5-HT + axons of descending raphespinal tract in epicenter of CCC vs. p<0.0001  dCCC: Increase of 5-HT + axons vs. CCC p<0.0001  Reduced 5HT+/synaptophysin+ axons indicating loss of descending serotonergic input in CCC vs. control p<0.0001  dCCC: Increases 5HT+/synaptophysin+ axons vs. CCC p<0.0001  Increased GAP43 (axonal sprouting) in all sites in the dCCC vs. control p<0.0001  Increased Iba+ in CCC above, below and at compressed site p<0.01, p<0.001, p<0.0001, respectively vs. control  dCCC reduces Iba+ above and at compressed site p<0.01, p<0.01 vs. CCC  Reduced GFAP (astrocytes) at epicenter of CCC vs. control p<0.01, increased GFAP above and below compressed site vs. control. (p<0.01, p<0.001, respectively). There was no change in the decompressed group.  Myelin changes:  CCC: Decreased fluoromyelin below the lesion site  dCCC: Decreased fluoromyelin above the compression site. | KF:  Spinal cord compression resulted in significant  locomotor deterioration, increased expression of the axonal injury marker APP, and loss of serotonergic fibres.  Surgical decompression partially reversed the deficits and attenuated APP expression. Decompression was also  associated with axonal sprouting, reflected in the restoration of serotonergic fibres and an increase of GAP43  expression. Promoting axonal plasticity may therefore be a therapeutic strategy for promoting neurological  recovery in DCM.  L: Fluoromyelin not very sensitive for detecting demyelination  Plasticity not tested at other levels such as the cortex and subcortical areas.  No cross validation of immunohistochemistry findings |
| Vidal et al (^57^) | Animals: 148 Adult C57BL/6 female mice (8 weeks old)  Groups:  (a) DCM with early sham decompression (DCM-E)    (b) DCM with early (6w) decompression (DCM-E + Dec)  (c) DCM with delayed sham decompression (DCM-D)  (d) DCM with delayed (12w) decompression (DCM-D + Dec)  Methods:  Aromatic polyether insertion C5-C6 to induce osteoid formation due to to the precipitation of inorganic salts.  Surgical decompression: Microdrill used to remove the fused laminae and relieve compression between the biopolymer and the laminae.  Sham-decompressed animals: Same surgical procedure as decompressed mice without decompression.  Direction of compression: Posteriorly  Duration of compression: 16 weeks | Compression ratios:  DCM-E + Dec 14.4% (n=5) vs DCM control 32% (n=5) *P <0.01*  DCM-D + Dec 12.8% vs DCM control 47% (*n* = 5) *P* < 0.05  Spinal cord blood flow (ml/min/100 g) after decompression:  DCM-E + Dec: 33.2 ± 20.8 (n=6) vs DCM-E 23.2 ±12.2 (n=7) vs naïve animals 42.1 ± 13.5 (n=6) ns  DCM-D + Dec 55.10 vs DCM-D 24.2 P<0.05  Cytokine profile following decompression  Early decompression  ELISA:  Increased levels of G-CSF, LIF, CCL-3 in DCM-E + Dec vs DCM-E p<0.05  IL-6, CXCL10, CCL-2 in DCM-E + Dec ***P* < 0.01  Increase in LIF, CCL-3, CCL-2, and CXCL10 that lasted for 5 weeks after surgery in DCM-D + Dec group vs DCM-D group.  Increased levels of G-CSF, IL-6, CXCL10, LIF, CCL-3, and CCL-2 in DCM-D + Dec (n=5-7) vs DCM-D (n=5) **P* < 0.05; ***P* < 0.01  Increased CXCL10, CCL-2 in DCM-D vs DCM-E (*P* < 0.01), and LIF (*P*< 0.05) at 24 hours after sham decompression.  Ratio of inflammatory/patrolling monocytes  Not significantly different between the DCM-E and DCM-E + Dec groups at all time points (24 hours [DCM-E, *n* = 9; DCM-E + Dec, *n* = 10], 2 weeks [DCM-E, *n* = 5; DCM-E + Dec, *n* = 6], and 5 weeks [DCM-E, *n* = 5; DCM-E + Dec, *n* = 9]).  Higher in DCM-D + Dec vs DCM-D at 2 and 5 weeks after decompression (DCM-D, *n* = 5; DCM-D + Dec, *n* = 5–8). ***P* < 0.01  Galectin-3  Increased in delayed decompression  WB:  DCM-D + Dec 1.2 (n=7) vs DCM-D 0.4 (n=5) ***P* < 0.01  DCM-E 0.4 (n = 5) vs DCM-E + Dec 0.5 (n = 5)  Astrogliosis  Attenuated in early decompression  GFAP immunoreactivity  GFAP Intdens  DCM-E + Dec 1x 10^7^ (*n* = 5) vs DCM-E 0.5x 10^7^ group (*n* = 5). **P* < 0.05  DCM-D 0.9 x 107 (n=5) vs DCM-D + Dec 2.1 x 10^7^ (n=5) ***P < 0.001  Gait deficits  Attenuated in early decompression  Forepaw swing speed (m/s) DCM-E + Dec 0.55 vs DCM- E 0.45, *P < 0.05  Hindpaw swing speed (m/s) DCM-E + Dec 0.55 vs DCM- E 0.48, *P < 0.05  Forepaw stride length (mm) DCM-E + Dec 65 vs DCM- E 52, *P < 0.05  Hindpaw stride length (mm) DCM-E + Dec 55 vs DCM- E 50, P = 0.13  Forepaw swing speed (m/s) DCM-D + Dec 0.48 vs DCM- 0.45, ns  Hindpaw swing speed (m/s) DCM-D + Dec 0.5 vs DCM- D 0.4, ns  Forepaw stride length (mm) DCM-D + Dec 58 vs DCM- D 58 , ns  Hindpaw stride length (mm) DCM-D + Dec 57 vs DCM- D 58, ns | KF:  Delay in decompression exacerbates the extent of ischemia-reperfusion injury and is associated with worse neurological outcome. |
